# Recent Advances in Understanding of the Etiopathogenesis, Diagnosis, and Management of Hair Loss Diseases

**DOI:** 10.3390/jcm12093259

**Published:** 2023-05-03

**Authors:** Misaki Kinoshita-Ise, Masahiro Fukuyama, Manabu Ohyama

**Affiliations:** Department of Dermatology, Kyorin University Faculty of Medicine, Tokyo 181-8611, Japan

**Keywords:** alopecia areata, androgenetic alopecia, female pattern hair loss, cicatricial alopecia, trichoscopy, JAK inhibitor

## Abstract

Hair-loss diseases comprise heterogenous conditions with respective pathophysiology and clinicopathological characteristics. Major breakthroughs in hair follicle biology and immunology have led to the elucidation of etiopathogenesis of non-scarring alopecia (e.g., alopecia areata, AA) and cicatricial alopecia (e.g., lichen planopilaris, LPP). High-throughput genetic analyses revealed molecular mechanism underlying the disease susceptibility of hair loss conditions, such as androgenetic alopecia (AGA) and female pattern hair loss (FPHL). Hair loss attracted public interest during the COVID-19 pandemic. The knowledge of hair loss diseases is robustly expanding and thus requires timely updates. In this review, the diagnostic and measurement methodologies applied to hair loss diseases are updated. Of note, novel criteria and classification/scoring systems published in the last decade are reviewed, highlighting their advantages over conventional ones. Emerging diagnostic techniques are itemized with clinical pearls enabling efficient utilization. Recent advances in understanding the etiopathogenesis and management for representative hair diseases, namely AGA, FPHL, AA, and major primary cicatricial alopecia, including LPP, are comprehensively summarized, focusing on causative factors, genetic predisposition, new disease entity, and novel therapeutic options. Lastly, the association between COVID-19 and hair loss is discussed to delineate telogen effluvium as the predominating pathomechanism accounting for this sequela.

## 1. Introduction

Hair loss diseases are heterogenous, consisting of conditions with respective pathophysiology and clinicopathological characteristics [1,2,3]. Despite their high prevalence, therapeutic options for hair loss diseases have still been limited when compared to other major dermatological problems, such as atopic dermatitis and psoriasis. To fully establish therapeutic strategies, accurate diagnosis and disease severity assessment are essential.

Recent progress in disease severity assessment and development of diagnostic techniques for hair loss diseases have been remarkable. One of the hardships in diagnosing hair diseases had been the paucity of globally standardized criteria/guidelines essential for the diagnosis of rare and/or complicated conditions [4,5]. In addition, measurement tools to score disease severity/activity and/or subgroup phenotypic variants had been limited, making clinical assessment and therapeutic planning challenging [6,7]. Recently invented diagnostic systems are useful for providing more reliable guidance than previous versions [4,7,8]. Recent studies/reviews refined or standardized diagnostic procedures represented by trichoscopy and hair pull test with evidence-based data and minimized the variance among practitioners [9,10,11]. Furthermore, the development of novel non-invasive diagnostic modalities is in progress [12,13].

In the last two decades, major breakthroughs have been made in hair follicle (HF) biology and immunology, represented by the identification of bulge stem cells and discovery of HF immune privilege (HF-IP) [14,15], enabling in-depth dissection of the pathomechanism underlying major forms of hair loss diseases such as IP-collapse in alopecia areata (AA) [1] and the loss of stem cells in primary cicatricial alopecias, including lichen planopilaris (LPP) [16]. Recent genetic and epidemiological analysis elucidated disease susceptibility for androgenetic alopecia (AGA) and female pattern hair loss (FPHL) [17] and revealed a possible link with environmental factors and frontal fibrosing alopecia (FFA) [18]. These discoveries shed light on the etiopathogenesis for each condition, providing rationales for therapeutic modalities, as the genome-wide association studies (GWAS) have led to the invention of Janus kinase inhibitor for severe AA [19].

Hair loss attracted public interest during the COVID-19 pandemic as a frequently observed sequela. A variety of hair diseases, such as AGA, AA and telogen effluvium (TE), have been implied as a possible pathomechanism [20], which deserves bibliographical consideration.

It is noteworthy that the knowledge of hair loss diseases is robustly expanding and thus requires timely updates. This narrative review attempts to comprehensively summarize recent advances in the understanding of the etiopathogenesis, diagnosis, and management of representative hair loss diseases. First, the latest diagnostic criteria and classification/soring systems and modalities for hair loss diseases are introduced, and then non-cicatricial and cicatricial alopecias are sequentially reviewed, focusing on the advances made in our understanding of their etiopathogenesis and development of treatment options.

## 2. Diagnosis and Severity Evaluation

The diagnosis of hair diseases can be made by the combination of standard procedures such as medical interview, physical examination, and laboratory examinations in combination with hair disease-specific diagnostic modalities represented by trichoscopy and a hair pull test. The following sections introduce current standards for the diagnosis and pattern/severity evaluation of hair diseases, along with updates and new insights.

### 2.1. Diagnostic Criteria and Classification/Scoring Systems

Table 1 summarizes the currently available diagnostic criteria and classification/scoring systems for each disease. Diagnostic criteria are not essential but beneficial for hair diseases which are rare or could otherwise be misdiagnosed.

For instance, FFA, a representative cicatricial alopecia and the variant of LPP, can be confused not only with “classical” LPP but also with AGA. Previously, no globally consented diagnostic criteria had been established, and thus the diagnosis had been often made based on clinicians’/researchers’ discretion or in locally accepted manners [21]. Recently, international guidelines for clinical trials of FFA were proposed by the international FFA cooperative group (IFFACG) [4] (Table 1a). These guidelines include diagnostic criteria (International FFA Cooperative Group Criteria for FFA), along with severity rating methods, and assessment measures, which were agreed upon by more than 90% of all IFFACG members on each item/recommendation. The diagnostic criteria present five common findings of FFA, each of which have 1 or 2 points, and ≥4/7 points are needed to confirm the diagnosis of classic FFA. They also provide criteria for probable FFA in which typical signs such as frontal hairline recession can be absent. Although these consensuses were made for the purpose of providing reliable standards to conduct clinical trials, they would be helpful for clinicians to diagnose FFA and evaluate its severity in daily practice.

Fibrosing alopecia in a pattern distribution (FAPD) is another rare condition in which features of LPP and AGA were concomitantly observed [22]. The diagnosis can be challenging due to its mixed phenotypic manifestation. Griggs et al. proposed the diagnostic criteria for FAPD in 2020 based on the outcome of the literature review of 15 articles [5] (Table 1b). The criteria are composed of seven major and six minor items with regards to clinical, trichoscopic, and histopathologic features, outlining this entity and solidifying the disease concept. How many criteria items should be satisfied to confirm the diagnosis remains elusive, and thus, further optimization is required to fully establish the diagnostic criteria for FAPD.

Classification/scoring systems are useful for subgroup disease phenotypes or activity/severity once the diagnoses are made. Classifications are used to categorize patterns or types of hair loss, while scoring systems are used for assessing the initial status of the disease and planning treatment approaches and predicting the responses to therapeutic interventions. Some systems can provide tools for both classification and scoring. The Severity of Alopecia Tool (SALT) I was originally developed for the assessment of AA but can also be useful in the severity assessment of other types of hair loss [23]. Its updated versions, SALT II [24] and III [4], have been published and can be adopted in particular situations requiring better precision (Table 1c). For AA, the severity can be graded into S0, no hair loss; S1, less than 25% hair loss; S2, 25–49% hair loss; S3, 50–74% hair loss; S4, 75–99% hair loss; and S5, 100% hair loss based on SALT [23] (Table 1d). Recently, a more practical AA severity scale was developed via an academic–industrial collaboration [25]. In this scale, AA is classified into three severities: mild (20% or less scalp hair loss), moderate (21–49% hair loss), and severe (50–100% hair loss). When one or more of the following factors are present, a severity rating is increased by one level in mild or moderate cases: (1) negative impact on psychosocial functioning resulting from AA, (2) noticeable involvement of eyebrows or eyelashes, (3) inadequate response after at least 6 months of treatment, and (4) diffuse (multifocal) positive hair pull test consistent with rapidly progressive AA (Table 1e).

The Hamilton–Norwood classification is the most disseminated classification/scoring system for AGA, in which the pattern and severity of hair loss are represented by seven categories (I to VII) and variants (e.g., III vertex) based on hair thinning of the vertex and recession of the frontal hair line [26] (Table 1f).

**Figure 1:**
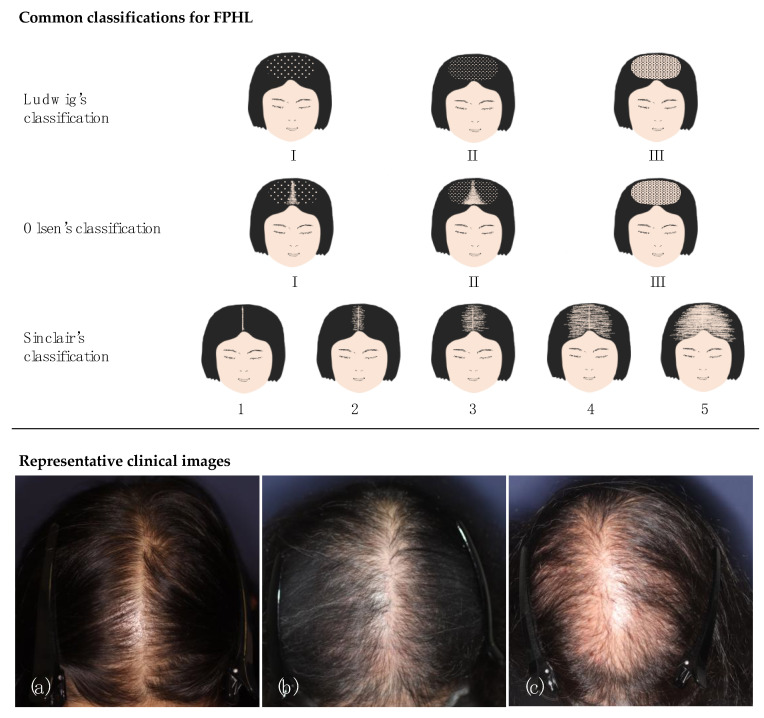
Common classifications for FPHL and representative clinical images. Ludwig’s, Olsen’s, and Sinclair’s classifications are comparatively presented. Image (**a**) does not demonstrate remarkable frontal accentuation and, thus, is better classified into Ludwig’s II or Sinclair’s 4. Image (**b**) can be more preferentially classified into Olsen’s II than into Ludwig’s II or Sinclair’s 3, considering frontal accentuation. Image (**c**) demonstrates mild hair thinning limited to the midline and, thus, is best classified into Sinclair’s 2.

Ludwig’s classification [27] subdivides FPHL cases based on the extent of hair thinning on the crown into three grades (Table 1h), and Olsen’s classification [28] focuses on the frontal accentuation other than diffuse hair thinning (Table 1i). Currently, Sinclair’s classification (Women’s Alopecia Severity Scale), which is a five-grade visual scale assessing midline hair density (the width of hair part), is more widely used in clinical practice [29,30] (Table 1j and Figure 1). By adopting a wider range of grades, Sinclair’s classification makes up for the shortcomings of Ludwig’s classification in which even the mildest category (Ludwig I) demonstrates clearly recognizable hair loss and thus would not be suitable for the distinction of mild FPHL. How to select the most suitable classification form among them for the severity evaluation of each case is illustrated in Figure 1a–c. A recent investigation reported that the “Trichoscopy Derived Sinclair Scale”, as calculated by the formula “3.9 × log (1/cumulative hair thickness density [total diameter of hair growing in scalp area unit]) as detected by trichoscopy) + 2.4”, enables a more precise evaluation [8] (Table 1k).

Recent publications proposed new scoring systems focusing on early stage FPHL. Based on the clinical photographs of 46 Japanese women with pattern hair loss, the severity grading system was proposed and consisted of six severities: Stage 1 (mildest) to Stage 6 (severest), in which the severity of Stage 6 is comparable to Sinclair 2 (the second mildest severity) [31] (Table 1l). Kaneko et al. took advantage of the surface-reflected light generated by camera flashlight on clinical picture recording and developed a grading scoring system [32] (Table 1m). The FPHL Severity Index (FPHL-SI), which was published in 2016 by nine world hair experts, was adjusted to evaluate the early stage FPHL without obviously visible hair loss [7]. This severity scale combines four validated measure items: the amount of hair shedding, midline hair density, and two trichoscopic findings (hair diameter diversity and the proportion of follicular units with single hair (increased in the affected area) or those triple hair (decreased compared to that in occiput)) [7] (Table 1n). As truly severe FPHL cases are rarely encountered, these approaches could provide more convenient evaluation tools for clinical use.

AGA and FPHL are influenced by racial predispositions, and thus ethnicity consideration may be required to categorize certain cohorts. Asian AGA can manifest distinctive hair loss limited to the vertex area with minimal recession of the frontal hair line, which would not be well classified by the Hamilton–Norwood classification [33]. The basic and specific (BASP) classification proposed by Lee et al. addressed this issue by involving a scoring scale for vertex hair loss [6]. This classification depicts the pattern and severity of hair loss by the combination of the basic (BA) types (L, M, C, and U) for the shape of the anterior hair line and specific (SP) types (V and F) for hair density in vertex and frontal areas, with the respective severity scores and facilitates elaborate description of AGA/FPHL state in Asians [31] (Table 1g).

Classification/scoring systems for cicatricial alopecia have been sparse. The LPP activity index (LPPAI) was introduced to evaluate the disease activity of LPP and FFA in 2010 [34] (Table 1o). For FFA, the FFA severity index (FFASI) and FFA severity score (FFASS) were proposed in 2016 [35] and 2018 [36], respectively (Table 1p,q), and the FFA global staging score was updated by IFFACG in 2021 to complement the limitations [4] (Table 1r).

**Table 1:** List of diagnostic criteria and classification/scoring systems for major hair loss diseases.

Hair Diseases	Diagnostic Criteria
Frontal fibrosing alopecia	(a) **International FFA Cooperative Group Criteria for FFA (International guidelines for clinical trials of FFA [4])**: Combination of clinical and pathological findings to diagnose classic FFA (Frontal hairline recession with loss of follicular ostia is necessary. Other items include positive biopsy result, eyebrow loss, perifollicular anterior scalp erythema, and perifollicular anterior scalp hyperkeratosis/scale) and probable FFA (frontal hairline recession is not necessary. Facial papules, preauricular hair loss, and absence of vellus hairs in affected hairline are added). Each item counts one or two points. Four or more points are needed to diagnose each condition.
Fibrosing alopecia in a pattern distribution	(b) **“Proposed diagnostic criteria for FAPD” [5]:** Criteria include seven major items (symmetric hair loss in androgen-dependent areas, sparing of androgen-independent areas, perifollicular casts/hyperkeratosis, loss of follicular ostia, hair density variability, lymphohistiocytic infiltrate around isthmus and infundibular region, and concentric perifollicular lamellar fibrosis) and six minor items (sparing of non-scalp hairs, perifollicular erythema, hair tufting, predominance of single hair follicles, interface dermatitis, and fibrosed follicular tracts) in three categories: clinical, trichoscopy, and histopathology.
	**Classification/scoring systems**
All hair loss diseases	(c) **SALT (I, II, and III) [4,24]**: Percentage of hair loss area (0–100) calculated by subdividing scalp surface area into four quadrants.
Alopecia areata	(d) **Severity scale [23]**: Severity subgrouping (S0–5) based on the SALT score: S0, no hair loss; S1, less than 25% hair loss; S2, 25–49% hair loss; S3, 50–74% hair loss; S4, 75–99% hair loss; and S5, 100%. (e) **Severity scale for clinical use [25]**: Mild AA, 20% or less scalp hair loss; moderate AA, 21–49% scalp hair loss; and severe AA, 50–100% scalp hair loss. If mild or moderate, increase AA severity rating by 1 level if 1 or more of the following is present: negative impact on psychosocial functioning resulting from AA, noticeable involvement of eyebrows or eyelashes, inadequate response after at least 6 months of treatment, and diffuse (multifocal) positive hair pull test consistent with rapidly progressive AA
Androgenetic alopecia	(f) **Hamilton–Norwood classification [26]**: The pattern and severity of hair loss is represented by seven categories (I to VII) and variants (e.g., III vertex) based on hair thinning of the vertex and recession of the frontal hair line.
	(g) **BASP classification [6]**: The scale of pattern and severity of hair loss are composed of a basic type (L, M, C, and U) and specific type (V and F).
Female pattern hair loss	
	(h) **Ludwig’s classification [27]**: severity scale based on degree of diffuse hair thinning on crown from I (mildest) to III (severest). (i) **Olsen’s classification [28]**: A severity scale of diffuse hair thinning on the crown (I to III), with the concept of frontal accentuation. (j) **Sinclair’s classification (Woman’s Alopecia Severity Scale [WASS]) [29]**: A severity scale from 1 (mildest) to 5 (severest) based on midline hair density. (k) **Trichoscopy Derived Sinclair Scale [8]**: A scale of hair density calculated by the formula of 3.9 × log (1/cumulative hair thickness density [total diameter of hair growing in scalp area unit]) as detected by trichoscopy) + 2.4. (l) **Shiseido’s classification [31]**: A grading system (Stages 1–6) based on the hair thinning on the crown. Stage 6 is comparable to Sinclair 2. (m) **Kaneko’s classification [32]**: A grading system (Grades 1–5) judged by the pattern of surface-reflected light, as detected by clinical picture recording. (n) **FPHL-SI [7]**: A severity scale for early FPHL (0–20) combining four measure items: the amount of hair shedding, midline hair density, and two trichoscopic findings.
Lichen planopilaris	(o) **LPPAI [34]**: An activity index (0–10) calculated by the formula including six symptoms (pruritus, pain, burning, erythema, perifollicular erythema, and perifollicular scale), the result of pull test, and the tendency of spreading.
Frontal fibrosing alopecia	(p) **FFASI [35]**: A severity index (0–100) combining the extent of recession of hair line in five anatomical parts, existence of hair loss in five body parts, and six additional features. (q) **FFASS [36]**: A severity score (0–25) by summing the extent of alopecia score (hair line recession and loss of eyebrows, 0–21) and grade of inflammation score (pruritus and pain, 0–4) (r) **Frontal fibrosing alopecia global staging score (international guidelines for clinical trials of FFA) [4]**: A staging system comprising five common findings: scalp hair loss, eyebrow loss, facial papules, prominent forehead veins, and facial hyperpigmentation, each of which have a range of numbers (S_0–4_E_0–2_P_0–1_V_0–1_H_0–1_).

BASP, basic and specific; FAPD, fibrosing alopecia in a pattern distribution; FFA, frontal fibrosing alopecia; FFASI, Frontal Fibrosing Alopecia Severity Index; FFASS, Frontal Fibrosing Alopecia Severity Score; FPHL-SI, Female Pattern Hair Loss Severity Index; LPPAI, Lichen Planopilaris Activity Index; SALT, Severity of Alopecia Tool.

### 2.2. Scalp and Hair Examination

Upon scalp and hair examination, it is crucial to clarify if the patient has either hair thinning, shedding, or both. Rarely, other symptoms, including an abnormal hair shaft morphology and remarkable increase of gray hairs, can be presented. Hair thinning can be evaluated by the criteria/classifications described above depending on the diagnosis. The hair pull test is the most popular technique to assess the presence and extent of hair shedding. The updated guidelines were proposed to minimize the limitations of the procedure in 2017 [11], and modifications for Asian- and afro-textured hairs were recently published as the supplement [10]. The assessment of the severity of hair shedding at a single medical consultation would hardly be possible, as the symptom can be influenced by multiple factors, such as hairstyle and daily haircare. Daily hair counts, the collection of shed hair by patients themselves, is helpful to semi-quantify hair shedding and to ascertain if the amount exceeds physiologic hair loss [37]. Other quantitative evaluation methodologies include the daily hair card [38], tag test [38], wash test (standardized and modified) [38], and contrasting felt examination [37].

### 2.3. Laboratory Examinations

Laboratory examinations can sometimes support the diagnosis of hair diseases. In particular, a blood test can be considered to search for the cause of TE. Although supporting evidence is rather insufficient, hypothyroidism/hyperthyroidism and renal and liver dysfunction can be preferentially examined. Serum zinc, iron, ferritin, vitamin D, and vitamin B12 can also be investigated [2]. One report suggested that vitamin B12 is associated with TE, accompanied with scalp dysesthesia [39]. For other hair diseases, the usefulness of blood tests has not been fully endorsed; however, abnormal results can be found in certain hair diseases. S1 guideline published by the European Consensus group described that a thyroid-stimulating hormone might be worth examining as laboratory investigation for FPHL associated diffuse hair shedding [40]. The guideline also concluded that an extensive endocrinological workup would not be essential unless medical history and physical examination are indicative of the presence of androgen excess (polycystic ovary syndrome, cycle disturbances, androgen-producing tumors, etc.) [40]. For cases with the signs of androgen excess, examinations of the free androgen index (FAI = total testosterone level/sex hormone-binding globulin level) and prolactin level were recommended [40]. Vitamin D deficiency/insufficiency has been reported to be associated with various hair diseases, including LPP/FFA, AGA, central centrifugal cicatricial alopecia (CCCA), and AA, along with TE. Patients with LPP had 8.3 times higher odds, and TE patients had 3.7 times higher odds of severe vitamin D deficiency when compared with AA patients [41]. Severe deficiencies of zinc, copper, or Vit B-complex deficiencies have been reported to be the causes of alopecia [42,43].

### 2.4. Trichoscopy

**Figure 2:**
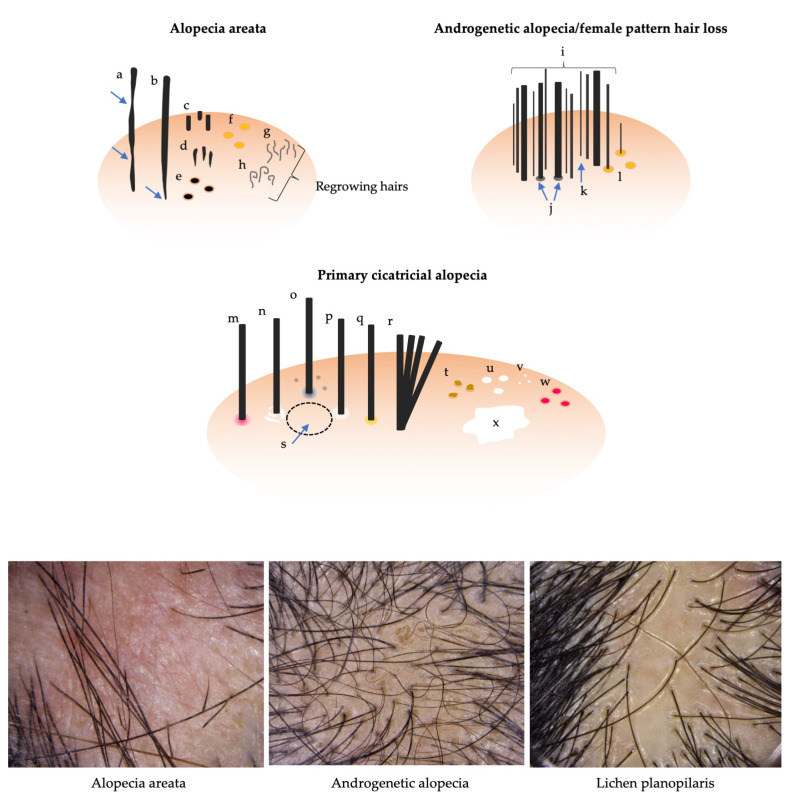
The schema of representative trichoscopic findings of hair loss diseases and trichoscopic images: (a) Pohl-Pinkus constrictions, (b) coudability hair (tapered hair), (c) broken hairs, (d) exclamation mark hairs (tapering hairs), (e) black dots, (f) yellow dots, (g) short vellus hairs, (h) pigtail hairs, (i) hair diameter diversity, (j) peripilar sign, (k) focal atrichia, (l) yellow dots (less prominent than alopecia areata), (m) perifollicular erythema, (n) perifollicular scales, (o) blue-grey dots (targetoid and speckled), (p) perifollicular whitish halo, (q) follicular pustule (more common in neutrophilic conditions), (r) tufted hairs (typical in folliculitis decalvans), (s) absence of follicular openings, (t) follicular keratotic pluggings (more common in discoid lupus erythematosus), (u) white dots, (v) pinpoint white dots, (w) follicular red dots (typical in discoid lupus erythematosus), and (x) white patch. Clinical images of alopecia areata with the findings of (c), (d), (e), and (g); androgenetic alopecia with the findings of (i); and lichen planopilaris with the findings of (n) and (s).

Trichoscopy is the most prevailing non-invasive diagnostic technique for the diagnosis of hair diseases. This technique was developed through the experience-based accumulation of diagnostically important or disease-specific findings. Currently, used findings can be grouped into changes in hair shaft, follicle and perifollicular area, scalp, and hair distribution pattern [9]. Respective findings in AA, AGA/FPHL, and primary cicatricial alopecia are displayed in Figure 2, and their definitions are summarized in Table 2 [9]. The paucity of the evidence-based guidelines/algorithms and resultant confusion in the terminology, even among experts, represent the limitation of trichoscopy [9]. A few flowchart diagnostic tools have been proposed [44,45,46]; however, these algorithms are not based on the objective data. Some systematic reviews and meta-analyses have been published for major hair diseases, such as AA [47], tinea capitis [48], primary cicatricial alopecia [49], discoid lupus erythematosus [50], and trichotillomania [51]. A recent review integrated terminology and proposed the flowchart algorithm based on a systematic approach [9]. This algorithm, as well as previous ones, suggest that trichoscopic assessment would better start with the differentiation of cicatricial and non-cicatricial alopecia by evaluating the presence or absence of follicular openings and then proceed to the detection of some of the more disease-specific or -sensitive findings, enabling the practitioners to make the diagnosis [9,44,45,46]. For example, AA can be diagnosed when either of black dots or broken hairs are detected, and then either coudability (tapered) hairs or exclamation-mark (tapering) hairs are found [9].

### 2.5. Other Non-Invasive Imaging Technologies

As trichoscopy detects changes observable on the scalp, other non-invasive modalities are needed to directly probe dermal-to-subcutaneous changes. Ultrasound, especially the one with high-frequent transducers, has been reported to be effective to diagnose/evaluate hair diseases [12,52]. This technique has a hidden potential to replace scalp biopsy by depicting clinically meaningful histological findings, such as the pattern and severity of inflammation and fibrosis, ratio of telogen/anagen hairs, and subcutaneous abnormalities [12]. Other emerging imaging techniques include optical coherence tomography [13,53,54], phototrichogram [55], videodermoscopy [56], and reflectance confocal microscopy [57,58], which have been reported to provide non-invasive and supportive diagnostic tools for hair and scalp diseases.

### 2.6. Scalp Biopsy

Currently, the scalp biopsy represents the most reliable diagnostic technique. To enhance diagnostic accuracy, the preparation of both vertical and transverse sections is recommended [59,60]. Vertical sections are used to evaluate interfollicular epidermal and dermal changes and analyze whole hair shafts/follicles at different depths, while transverse sections provide the information of hair cycle status, degree of hair miniaturization, types of perifollicular inflammation and fibrosis, etc. The average number of total hairs, the ratio of vellus/terminal hairs, and the ratio of telogen/anagen hairs in different racial cohorts have been reported in previous studies [61,62,63,64,65]. According to these studies, Caucasians tend to present more follicular unit structures and a larger number of HFs than African and Asian people [62,64,65], while Asian people present with a greater ratio of terminal to vellus hairs than Caucasians and African people [62,64,65].

## 3. Non-Cicatricial Alopecia

### 3.1. Androgenetic Alopecia and Female Pattern Hair Loss

#### 3.1.1. Etiopathogenesis

AGA is one of the common hair loss conditions characterized by the miniaturization of HFs in the frontal and parietal regions of the scalp predominantly affecting prime-aged male [66]. The condition is with genetic predisposition, and the conversion of testosterone to dihydrotestosterone, a more active form, within the dermal papilla at the root of the HF by 5-α reductase has been shown to contribute to the etiopathology. In this condition, acceleration of the hair cycle has been conceived to account for hair follicular miniaturization and an increased ratio of kenogen HFs [67]. The involvement of the prostaglandin pathway [68], Wnt/β-catenin pathway [69], mitochondrial activity [70], and oxidative stress [71] has also been suggested. Recently, the association between the peroxisome-proliferator-activated receptor (PPAR) signaling pathway and HF miniaturization has been elucidated [72]. Upregulation of *PPARGC1α* in the inner and outer root sheath of HFs from AGA patients was implicated in the follicular miniaturization and eventually contributes to the development of characteristic clinical presentation [72]. Another recent investigation also suggested that the impairment of autophagy might take part in the etiopathogenesis of AGA [73]. These novel discoveries potentially set the basis for targets for the development of future therapeutic approaches.

Individual lifestyle and environmental factors may influence the onset and/or exacerbation of AGA [74]. A community-based sociomedical study found a statistically positive association between the risk for AGA and metabolic syndrome (odds ratio (OR), 1.67; 95% confidence interval (CI), 1.01–2.74; *p* = 0.04) and high-density lipoprotein cholesterol (OR, 2.36; 95% CI, 1.41–3.95; *p* = 0.001) [74]. Excessive free-radical generation induced by smoking and the environmental exposure to external insults, such as ultraviolet radiation, pollutants, chemical irritants, and microbes, have also been implicated in AGA etiopathogenesis [71]. In aggregate, reconsideration of the living environment and one’s lifestyle constitute important elements in the management of AGA that might have not been sufficiently emphasized previously.

**Figure 3:**
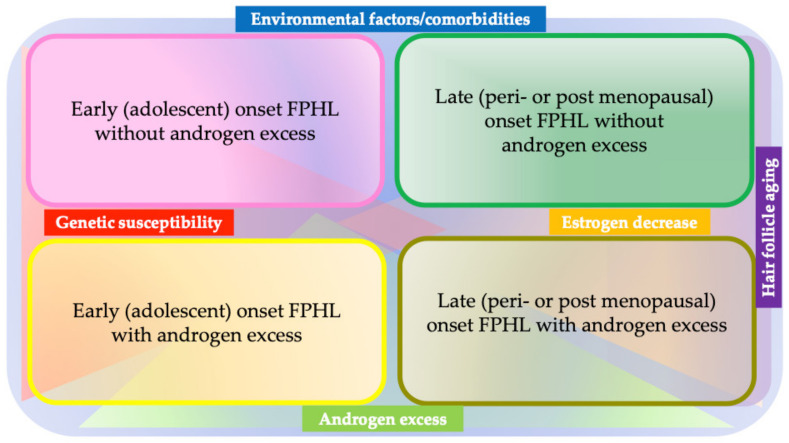
The heterogeneity of female pattern hair loss.

Unlike in AGA, the role of androgens in FPHL remains unclear, as the condition can be developed in individuals with normal androgen levels [75], and thus, the term “FPHL” is currently preferred over “female AGA” [76]. Genetic predisposition may play a role in the development of FPHL in such individuals. Indeed, a family history of FPHL has been reported in 40–54% of early onset cases with normal androgen levels [77]. A single SNP in AGA susceptibility locus on androgen receptor (AR)/ectodysplasin A2 receptor (EDA2R) has been shown to be associated with FPHL in UK patients, but not in a German patient group [17]. Another AGA-associated locus, 20p11, was not shown to be associated with FPHL [17]. Interestingly, a recent study from a Korean group [78] found that early onset FPHL (the onset at earlier than age 39) patients manifested clinical signs of hyperandrogenism, such as an excess of scalp sebum, folliculitis, hypertrichosis, and the presence of polycystic ovary syndrome and an association with a single nucleotide polymorphism of *PPARRGC1A*, *ABCC4*, *FSHB*, and *CYP19A*. Furthermore, the upregulation of PGC-1α, a gene product of *PPARRGC1A*, significantly suppressed hair growth in an organ culture model, suggesting a role of genetic predisposition in the etiopathogenesis of early onset FPHL [78]. Based on these findings and physical and laboratory examinations, FPHL is currently considered to be heterogenous, which has been proposed to be divided into four subsets depending on the age range and the presence or absence of androgen excess: early (adolescent) onset FPHL, with or without androgen excess; and late (peri- or post-menopausal) onset FPHL, with or without androgen excess (Figure 3) [79].

The incidence of FPHL tends to be higher in postmenopausal women with or without androgen excess [75]. In line with this, hair loss can be observed in ovariectomized mice with low estrogen levels, which can be prevented by the addition of exogenous estrogen, further emphasizing a possible contribution of low estrogen to the development of postmenopausal FPHL [80]. Intriguingly, angiopietin-2 (ANGPT2) was found to be located at the downstream of estrogen in mice, thus potentially providing a future therapeutic modality for FPHL [81].

The contribution of “microinflammation”, which refers to histopathological peri-infundibular lymphocytic cell infiltrates, has been suggested in AGA and FPHL pathophysiology [82]. Putative triggers include ultraviolet radiation, environmental pollutants, and perifollicular microbiota [75]. In line with this, a recent study demonstrated an increase in the abundance of *Cutibacterium acnes* in miniaturized HFs in AGA. The role of perifollicular dysbiosis in FPHL awaits further investigation [83].

Environmental (habitual and social) factors and comorbidities may also influence or be associated with etiopathogenesis and clinical manifestation in FPHL. Psychological stress, higher income, lack of physical activity, insufficient photoprotection, multiple marriages, smoking, hypertension, and diabetes mellitus have been related to FHPL. Of note, advanced-stage FPHL has been reported to be associated with aging, menopausal state, and hypertension, whereas acne vulgaris is rather related to the early stage [84]. Both hirsutism and acne vulgaris were more commonly seen in the Ludwig- and Hamilton–Norwood-type than in those with Olsen-type hair loss, and hypertension was frequent in the Ludwig type [84]. These observations further support that FPHL consists of heterogenous patient subgroups and emphasize the necessity of subtype-wise analysis for further dissection of the etiopathogenesis.

#### 3.1.2. Treatment

##### 5-α reductase Inhibitors

Oral 5-α reductase inhibitors, finasteride and dutasteride, have been widely prescribed for AGA [66]. Finasteride inhibits 5-α reductase type II, while dutasteride inhibits both type I and II isoforms [66]. A recent network meta-analysis demonstrated that the increase in total hair count at 24 weeks with 0.5 mg/day of dutasteride was more efficacious compared to that with 1 mg/day finasteride (mean difference (MD), 7.1 hairs/cm^2^; 95% CI, 5.1–9.3 hairs/cm^2^) with the similar adverse events profile [85,86]. Furthermore, a Korean group analyzed the long-term efficacy and safety profiles of finasteride and dutasteride; they reported that dutasteride-treated patients showed more significant improvement in hair growth compared to finasteride-treated patients (95% CI, 1.08–3.95; *p* = 0.029), and both medications were analogously safe (possibly with a lower incidence of sexual adverse events than previously reported; dutasteride = 1.6%, and finasteride = 1.1%) [87]. Accordingly, oral 5-α reductase inhibitors represent the first-line treatment modality for AGA because of their effectiveness and tolerability, and dutasteride further widened therapeutic options [66,87].

The use of oral 5-α reductase inhibitors in women is not approved by the FDA, and they are contraindicated for pregnant women because of their teratogenicity in the male fetus [75]. Several reports described the efficacy of oral finasteride for FPHL; however, the outcomes have been variable [75]. Considering the observation that oral finasteride (1.25 mg daily) was effective for FPHL patients with hyperandrogenism but not for those without that condition [88] and that oral finasteride at a different dose (2.5 mg daily) was more efficacious in improving hair loss in those with a lower Ludwig score and/or older onset [89], the reported outcomes could have been influenced by the way patients were recruited, as FPHL consists of heterogenous patient subsets with respective pathophysiology. Oral dutasteride has been reported to improve FPHL, as well [90]; considering the potential risk, off-label use for FPHL should require careful risk–benefit consideration.

In recent years, topical finasteride has been attracting great interest as a therapeutic modality for AGA and FPHL, and a review of the literature supported its safety profile and promise [91]. Still, the data are preliminary, and formulations, especially the finasteride concentration and the composition of the vehicle, have not been standardized [91]. The plasma dihydrotestosterone level can be moderately reduced [92], and therefore, safety concerns, especially potential teratogenicity, need to be addressed when used for FPHL.

##### Minoxidil

Topical minoxidil treatment is still a popular medication for AGA. Based on the results of previously conducted randomized controlled trials, 2% and 5% topical minoxidil formulas were approved by the Food and Drug Administration (FDA), and 1% and 5% formulas were available as an over-the-counter medicine in Japan [66,86]. For FPHL, topical minoxidil 2% solution twice daily and 5% minoxidil foam once daily were approved for FPHL by the FDA [75]. Both formulas have been reported to exhibit similar efficacy for FPHL [93]. The precise mechanism underlying the mode-of-action of minoxidil remains elusive. Minoxidil has been reported to promote dermal papilla vascularization via a stimulation of vascular endothelial growth factor (VEGF) expression, resulting in the maintenance of vascularization around anagen HF [94]. Furthermore, minoxidil is converted into its active form, minoxidil sulfate, by sulfotransferase expressed in the outer root sheath which opens potassium channels, resulting in the promotion of hair growth [95]. Previous reports suggested that sulfotransferase activity in the outer root sheath of plucked HFs can be considered a useful biomarker for predicting the response to topical minoxidil in AGA [95,96].

Oral minoxidil has been prescribed for both AGA and FPHL in some clinics [75]. Off-label use of oral minoxidil can increase hair density; however, it can also cause adverse events, such as postural hypotension, fluid retention, and hypertrichosis (facial hypertrichosis can be quite problematic for female patients) [75]. Indeed, the latest version of the Japanese Dermatological Association (JDA) guideline for the diagnosis and treatment of AGA and FPHL does not recommend the use of oral minoxidil [66]. However, in recent years, low-dose oral minoxidil is attracting interest as an effective and well-tolerated therapeutic option [97]. A recent multicenter study concluded that low-dose oral minoxidil had a good safety profile as a therapeutic option for hair loss diseases, including AGA and FPHL, with a discontinuation rate as low as 1.7% due to adverse events [97]. Low-dose oral minoxidil (0.25 mg daily) could be combined with an androgen antagonist spironolactone (25 mg daily) and used for FPHL treatment; this has been reported to be both effective and safe [98]. Larger placebo-controlled studies are required to convincingly justify the use of low-dose oral minoxidil for FPHL.

##### Platelet-Rich Plasma

Platelet-rich plasma (PRP) refers to autologous plasma containing highly concentrated platelets and their associated growth factors, including EGF, IGF-1, and VEGF, the factors known to play pivotal roles in regulating hair growth [99]. A previous systematic review and meta-analysis of 30 studies including 10 randomized controlled clinical trials reported that PRP treatment resulted in a significant increase in hair density (the mean increase in hairs/cm^2^ after PRP treatment was 33/cm^2^, which was a 20% increase from the baseline) and hair thickness (the mean increase in hair diameter was 32 μm, which was a 49% increase from the baseline) in male and female AGA [100]. Of note, recent studies showed that the combination of PRP and other modalities, including topical minoxidil and oral finasteride, synergistically promoted hair growth; the increase in hair density achieved by the combination of PRP and topical minoxidil was 1.74 and 2.9 times greater than those with topical minoxidil monotherapy and PRP monotherapy, respectively [101]. In addition, the mean terminal hair density 6 months after the combination of PRP and topical minoxidil or oral finasteride showed statistically greater improvement than monotherapy of topical minoxidil or oral finasteride (mean ± SD; 136.8 ± 44.1 hairs/cm^2^ versus 152.9 ± 44.6 hairs/cm^2^, *p* < 0.05) [102]. Thus, PRP with or without the other modalities could be a favorable therapeutic option, although the standardized protocol has not been fully established.

Intriguingly, in a randomized clinical trial where PRP or saline was intradermally injected into each side of the affected scalp, improvement of hair density was observed both in the PRP- and saline-treated area, presumably because of PRP diffusion [99]. PRP injection can be a fairly safe procedure; however, further accumulation of data and standardization of the protocol are required to fully support its use for AGA/FPHL.

##### Spironolactone and Other Hormone-Modulating Therapies

Spironolactone has been used as a therapeutic modality for FPHL. It acts as an aldosterone receptor antagonist and is a widely used diuretic for the treatment of various systemic conditions, including heart failure, hypertension, edema, and hypokalemia [103]. It is also known to antagonize the androgen receptor and therefore has been used for androgen-dependent conditions, including hirsutism, acne, hidradenitis suppurativa, and FPHL, particularly with the sign of hyperandrogenism, as an off-label medication in dermatological practice [104]. For FPHL, oral spironolactone at the dose of 50–200 mg/day is conventionally prescribed [105]. A recent retrospective and observational study including 79 women demonstrated that all patients maintained or improved their initial Sinclair score, with an average overall change of 0.65 after 6 months or longer use of the medication [106]. In addition, the effectiveness was independent of the concomitant use of other hair loss therapies, contraception, or menopause status. This observation supported the idea that spironolactone is an effective and well-tolerated therapeutic option for FPHL, either as monotherapy or adjunct therapy [106]. As mentioned above, combination therapy with low-dose minoxidil and spironolactone can also be safe and efficacious [98]. Topical spironolactone (1~5%), in combination with topical minoxidil, has been reported to achieve favorable outcomes [107,108].

The well-known side effects of spironolactone are hyperkalemia, agranulocytosis, cycle disturbance, breast tenderness, and hypotension; thus, regular monitoring of blood pressure and a blood test are recommended [104,105]. The potential risk of estrogen-dependent malignancies has also been debated. A recent retrospective analysis using an insurance database concluded that there is no association between the use of spironolactone and increased recurrence of breast cancer in female patients [109]; however, studies with a high level of evidence are limited, and careful consideration and follow-up are indispensable when the medication is used for those with medical/family history of estrogen-dependent malignancies.

Oral cyproterone acetate combined with ethinyl estradiol and oral flutamide are other conventional hormone-modulating therapies for FPHL, but supporting evidence is insufficient [79,110,111]. A recent study concluded that the efficacy of topical 17-αestradiol was inferior to that of finasteride 0.5% in combination with minoxidil 2% in postmenopausal FPHL [112].

##### Hair Transplantation

Hair transplantation is an established procedure to treat AGA and FPHL which is supported by the JDA guideline as a possible therapeutic option [66]. Insufficiency of donor site can be encountered in female patients [75].

##### Other Modalities

Lines of evidence supported the efficacy of low-level laser therapy (LLLT) for AGA and FPHL. LLLT has been reported to improve hair density, count, and growth [113]. Particularly, the LLLT utilizing the device irradiating the light wavelengths between 630 and 660 nm can be an alternative treatment modality for AGA [114]. The JDA guideline lists LLLT as a possible treatment modality for AGA/FPHL [66]. A 1555 nm fractional erbium-glass laser can also be used for FPHL [115]. LLLT has been considered to increase adenosine triphosphate production; modulate reactive oxygen species; and induce transcription factors, including nuclear factor kappa B and hypoxia-inducible factor-1, which are involved in cell proliferation and migration [114]. Because high-level evidence supporting the efficacy of LLLT is still insufficient [116], this modality could rather be a future therapeutic option.

Cetirizine, a selective H1-receptor antagonist, was recently shown to have anti-inflammatory effects via the inhibition of prostaglandin D2, which suppresses hair growth and induces the miniaturization of HF [117]. A systematic review suggested that 1% topical cetirizine could significantly improve total hair density (MD, 27.72; 95% CI, 26.68–28.76) and hair diameter (MD, 1.47; 95% CI, 1.22–1.72) compared with a placebo, and its efficacy might be equivalent to topical minoxidil in terms of improving hair diameter in AGA [118]. One-percent topical cetirizine may be useful to improve hair loss, especially in those insufficiently responding to topical minoxidil [118].

Intriguingly, a randomized placebo-controlled clinical study was conducted to assess the usefulness of autologous-cell-based therapy in which dermal sheath cup cells (DSCCs) were transplanted into the affected areas of AGA or FPHL. The study demonstrated that autologous-cell-based therapy for AGA and FPHL adopting intralesional injection of cultured DSCCs harvested from a non-affected occipital area increased the total hair density and cumulative hair diameter [119]. The safety and efficacy of this approach needs to be further evaluated by phase II/III clinical studies; however, the autologous-cell-based therapy may provide a strategy for treating pattern hair loss, especially FPHL.

In addition to the therapeutic modalities described above, various approaches, such as topical ketoconazole [120] and melatonin [121], nutritional supplementation [122], microneedling [123], and growth factor/mesenchymal stem cell injection [124], have been conceived and attempted to treat AGA/FPHL, with variable outcomes, and therefore further investigation is needed.

### 3.2. Alopecia Areata

#### 3.2.1. Etiopathogenesis

**Figure 4:**
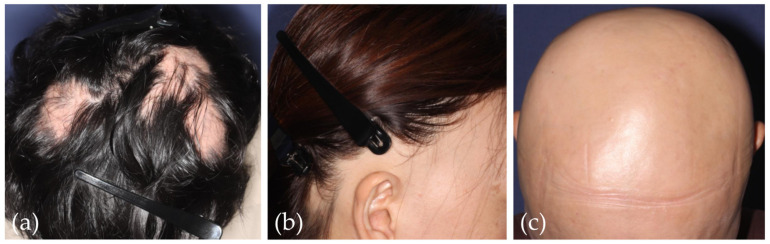
Representative clinical phenotypes of alopecia areata. (**a**) Typical form of alopecia areata presenting multiple alopecic patches, (**b**) alopecia ophiasis, and (**c**) alopecia totalis/universalis.

AA is a non-scarring hair loss disorder characterized by autoimmunity targeting anagen HFs [125]. Clinically, AA manifests several phenotypes: it typically presents a single alopecic patch or multiple alopecic patches on the scalp (Figure 4a) but occasionally demonstrates band-like hair loss along the hairline on the occipital and/or temporal region (alopecia ophiasis) (Figure 4b) and total scalp hair loss with or without whole-body hair loss (alopecia totalis/universalis) (Figure 4c) [1]. Rare manifestations include an ophiasis inversus/sisaipho pattern and a diffuse pattern [126]. The estimated lifetime prevalence has been reported to be around 1.45–2.18% [127,128,129]; therefore, AA is a relatively common disorder. Major advances have been made in our understanding of the etiopathogenesis of AA in which NKG2D+CD8+ cytotoxic T lymphocytes play major roles [130,131]. Multiple factors, such as genetic predisposition, atopic, and/or autoimmune background, in combination with triggering factors such as viral infection, local trauma, daily lifestyle, and physical or emotional stress, have been reported to play roles in the etiopathogenesis.

A surge of interferon (IFN)-γ, secondary to the aforementioned insults, has been proposed to elicit the collapse of HF-IP and subsequent autoimmune-response-targeting HF autoantigens best represented by the activation of autoreactive cytotoxic NKG2D+CD8+ T cells plays central roles in AA pathogenesis [1,130,131,132]. Furthermore, IFN-γ also induces the production of interleukin (IL)-2 and IL-15 from follicular epithelium, which further promotes the activation of CD8+ T cells to produce IFN-γ secretion, resulting in the formation of IFN-γ/IL-15 loop in persistent AA [132]. The JAK-STAT signaling pathway is located at downstream of these cytokines and is shown to be responsible for the prolongation of AA; based on this, JAK inhibitors were invented as the novel therapeutic agents for severe AA [133].

These immunoreactions are mainly attributed to type 1 immunity, as supported by the findings obtained by GWAS, global gene expression analyses, and experimentations using AA model mouse [1,132,134]. At the same time, recent studies also implied a possible contribution of type 2 immune response and Th17 axis to the etiopathogenesis of AA [135,136,137]. In addition, the influence of gut microbiota has been implicated in AA development, highlighting the complexity of AA etiopathology [138].

#### 3.2.2. Treatment

##### Optimization of Conventional Therapeutic Approaches

**Figure 5:**
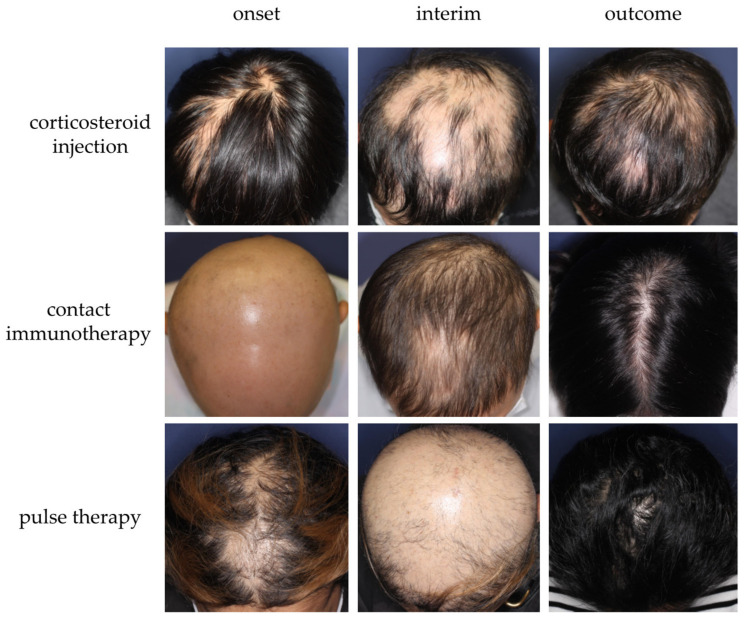
Representative clinical courses of alopecia areata treated by corticosteroid injection, contact immunotherapy, and intravenous corticosteroid pulse therapy (pulse therapy). Each patient is considered a good responder.

**Figure 6:**
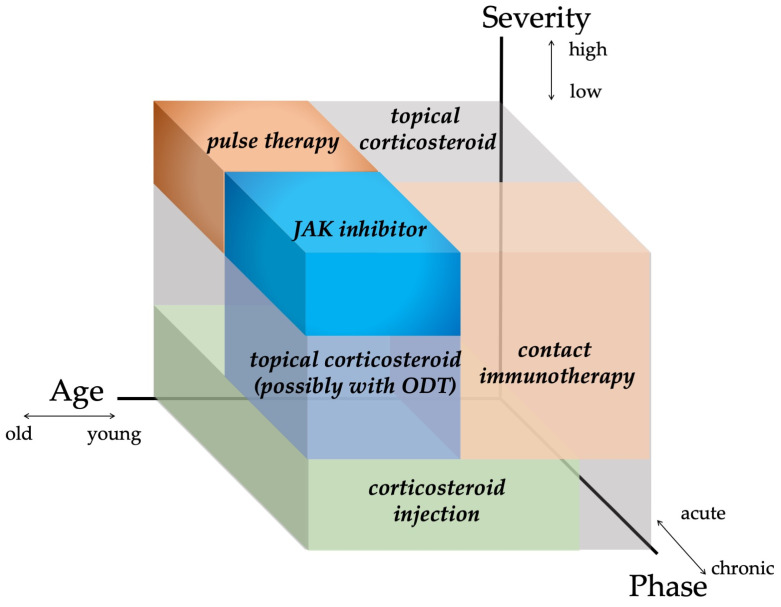
“Modified-AA-cube”, a schema illustrating a strategy for selecting a therapeutic approach in AA after the emergence of JAK inhibitor in market.

**Figure 7:**
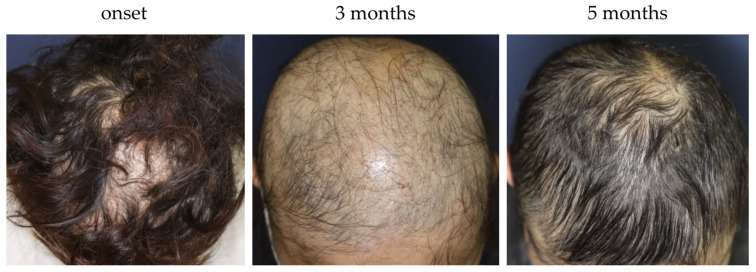
Typical clinical course of self-healing acute diffuse and total alopecia (sADTA). Despite rapidly progressive diffuse hair loss, A sADTA patient spontaneously improves without any intervention.

Several treatment modalities, including immunosuppressants (e.g., topical corticosteroid, corticosteroid injection, intravenous or oral corticosteroid pulse therapy, and oral methotrexate) or immunomodulating agents (e.g., contact immunotherapy), have been adopted to treat AA in recent years (Figure 5) [1]. For better management of AA using those modalities, optimization is pivotal. Several guidelines/expert consensuses have been published to date [1,126,139,140,141]. Those reported by Italian, Brazilian, and Australian experts narratively describe treatment options and provide flowcharts or algorithms for AA management [126,139,140], whereas the latest Japanese and British guidelines list respective treatments, along with the strength of recommendations and levels of evidence for each therapeutic modality [1,141]. In 2020, the international expert opinion on treatment for AA was summarized and published as the AA Consensus of Experts (ACE) [142]. In this report, a consensus was made on the use of topical corticosteroids as a first-line treatment [142]. The ranking or importance of other therapeutic modalities can be influenced by multiple factors, such as regional, racial, and cultural backgrounds, and more specifically, medical insurance systems [142]. For instance, the latest JDA guideline recommends intravenous corticosteroid pulse therapy as a possible therapeutic option for rapidly progressive AA with SALT score ≥50 and the duration of 6 months or less [1], whereas other guidelines/expert consensus do not positively support the use of it [126,139,140]. This suggests that the establishment of a globally consented flowchart/algorithm of AA management can be challenging. In principle, an AA management plan can be made in consideration of three major factors: age, severity (the size of affected area), and disease activity (acute or chronic) [1,126,139,140,141]. We recently proposed a three-dimensional diagram, namely the “AA-cube”, globally illustrating the strategy for treatment selection for AA (Figure 6) and a therapeutic flowchart for rapidly progressive subtype of AA [1]. Regardless of phenotypical severity, course observation can be a possible management plan for some AA cases. Patients with a relatively small number of AA patches frequently experience spontaneous remission within 1 year [140]. In addition, our case series study demonstrated that even a severe subset of rapidly progressive and diffuse AA cases could achieve almost full hair regrowth without any treatment, within a year, and can be named as self-healing acute diffuse and total AA (sADTA) (Figure 7) [143]. A case can be sADTA when the it has four or more of the following factors: being female, the absence of scalp pain and itch, the absence of extra-scalp hair loss, the predominance of club hair in the hair pull test, the predominance of short vellus hairs, and the increase in vacant follicular ostia all over the scalp, as detected by trichoscopy. A possible sADTA can be followed up for 3 or 4 months without interventions such as intravenous corticosteroid pulse therapy [143].

##### Janus Kinase (JAK) Inhibitor

As mentioned above, recent investigations elucidated that the JAK-STAT pathway plays a crucial role in the pathogenesis of AA [132]. The efficacy of the JAK inhibitor for moderate-to-severe AA has been supported by the results of several case series, as well as by ongoing international clinical trials (Table 3) [144,145,146,147,148,149,150]. The refinement of JAK inhibitors is in progress. The first-generation JAK inhibitors block more than one JAK family member, and the second generation of those specifically affects a single JAK family isoform [144]. Baricitinib, one of the representative first-generation JAK inhibitors blocking JAK 1 and 2, was approved by the FDA, the European Medicines Agency, and the Pharmaceutical and Medical Devices Agency in Japan, based on the results of two phase-three trials named BRAVE-AA1 (phase-three part) and BRAVE-AA2 [133]. In these trials, a total of 1200 patients were randomly assigned in a 3:2:2 ratio to take baricitinib at a dose of 4 mg, 2 mg, and placebo, and the percentage of patients who achieved a SALT score of 20 or less after 36 weeks of treatment was reported to be 38.8%, 22.8%, and 6.2% in BRAVE-AA1 and 35.9%, 19.4%, and 3.3% in BRAVE-AA2, respectively [133]. Adverse events represented by hyperlipidemia (34.8%), upper respiratory infection (7.0%), acne/folliculitis (5.3%), elevated levels of creatine kinase (4.5%), urinary tract infection (3.5%), and herpes zoster (1.0%) have been reported [133]; however, the incidence of major adverse events was quite low in these trials, and therefore, baricitinib for severe AA has been considered to relatively safe and, thus, tolerable. In those reports, the influence of discontinuation of baricitinib on responders was not described. Previous studies reported that the discontinuation of JAK inhibitors could lead to the recurrence of AA at the rate of 17.9–31.3% [151]. Furthermore, exacerbation of AA during the treatment has also been reported [152]. To date, phase-three clinical trials of other JAK inhibitors, including CTP-543 (deuterated form of ruxolitinib, JAK1/2 inhibitor) and ritlecitinib (JAK 3 inhibitor), are ongoing [144]. Additional JAK inhibitors are expected to be assessed for their efficacy regarding AA to widen the choice of therapeutic options.

##### Dupilumab

Dupilumab is a humanized monoclonal-antibody-blocking IL-4/13 receptor, which has been widely used to treat moderate-to-severe AD. Current studies implied that the administration of dupilumab is a possible therapeutic option for AA concomitant with AD [153,154,155]. Indeed, a phase 2a randomized clinical trial was conducted to evaluate the efficacy of dupilumab for AA [156]. In this trial, 32.5%, 22.5%, and 15% of dupilumab-treated patients (mean SALT score was 70.5, and mean eczema area and severity index (EASI) was 13.6 at baseline) achieved SALT_30_, SALT_50_, and SALT_75_, respectively, after 48 weeks of dupilumab therapy, while placebo-treated patients tended to have worsened SALT scores [156]. Furthermore, the patients with serum levels of IgE ≥200 IU/mL demonstrated a 6.25 times higher SALT_75_ response rate than those with IgE <200 IU/mL after week 72, and dupilumab responders were more likely to have an AD history and/or high serum levels of IgE compared to non-responders [156]. Another study revealed that AA patients with AD and high serum levels of IgE showed clinically severer forms of AA compared to those without AD and with normal IgE levels [157]. These findings suggested that dupilumab may be effective for AA patients with atopic background and that IgE level may predict its efficacy regarding AA in such patients. Another study showed that dupilumab-treated patients showed significant downregulation of type-2-related biomarkers represented by CCL13/MCP-4 and CCL18/PARC, associated with significant upregulation of hair keratins [158]. In contrast, the exacerbation or new onset of AA in dupilumab-treated AD patients has also been reported [159]. The most recent case series study reported insufficient improvement of AA even in the patients with higher IgE levels [57]. Thus, the efficacy of dupilumab for AA remains controversial. The distinct difference in the response to dupilumab in AA patients with AD could be explained by the characteristics of comorbid AD; Kageyama et al. reported that dupilumab would not be effective for AA with intrinsic AD but for those with extrinsic AD [160]. It should be noted that dupilumab is not approved for AA by FDA and would not be covered by the health insurance in Japan and in other countries [1].

##### Platelet-Rich Plasma

PRP holds promise as a novel therapeutic modality for AA [161]. The mechanism by which PRP exhibits its efficacy for AA remains elusive; the presence of TGF-β as an ingredient and suppression of MCP-1 may lead to an anti-inflammatory effect [161]. A systematic review and meta-analysis of four randomized clinical trials demonstrated that the decrease in the SALT score of PRP-treated AA patients was comparable to that in triamcinolone-acetonide-injection-treated groups [162]. The adverse events were relatively mild, including headache, itch, transient erythema, and edema [161]. Previous studies reported that a higher number of patients complained about pain during PRP administration than those subjected to triamcinolone acetonide injection [163,164,165]. An optimized treatment protocol has yet to be determined. A higher level of evidence is needed to fully establish PRP as a therapeutic option for AA.

##### Other Modalities

Statins have been expected to be a therapeutic option for AA [166]. Simvastatin has been reported to exhibit anti-inflammatory activities, including the modulation of JAK/STAT pathway, leading to the downregulation of type 1 cytokine response and inhibition of leukocyte function, and activate the Wnt/β-catenin signaling pathway [167]. Furthermore, a recent study showed that statins effectively repressed pathogenic NKG2D-MICA interactions [168]. The combination of simvastatin and ezetimibe has been shown to be effective to AA both in human and mouse disease model studies; 12 of 19 patients of AA with 40–70% scalp involvement showed >50% hair regrowth at week 24 [169]. In addition, simvastatin/ezetimibe intraperitoneal injection resulted in remission of AA lesion in C3H/HeJ mice [166]. Several biologics, including TNF-α blockers and monoclonal antibodies against IFN-γ, IL-12/23, and CD11, have also been tested for their therapeutic potential to AA [170,171,172,173]; however, their efficacy has not yet been established in the context of AA [1]. Apremilast, which is a phosphodiesterase 4 (PDE4) inhibitor and suppresses the production of pro-inflammatory mediators represented by IFN-γ via the upregulation of cAMP, had been expected to be useful for AA; however, a randomized placebo-controlled study negatively evaluated its efficacy in moderate-to-severe AA (Table 3) [174].

**Table 3:** List of clinical trials using JAK inhibitors and a phosphodiesterase 4 inhibitor to treat moderate-to-severe alopecia areata.

Drug	Study	Patient	Dose	Outcome
Tofacitinib (pan-JAK) [148]	Phase 2, open label	12 adult patients with moderate to severe AA	5 mg to 10 mg twice daily for 6 months	91% of the patients achieved SALT score improvement, and 67% patients reached SALT 50.
Ruxolitinib (JAK 1/2) [146]	Phase 2, open label	12 adult patients with moderate to severe AA	20 mg twice daily for 12–24 weeks	75% of the patients were responsive with the average of 92% hair regrowth.
Delgocitinib ointment [149]	Phase 2, double blind	31 adult patients with moderate to severe AA	30 mg/g ointment applied twice daily or vehicle control for 12 weeks	The mean decrease in SALT was 3.8 in the delgocitinib group and 3.4 in the vehicle group.
Ritlecitinib (JAK 3) [144]	Phase 2B/3, double blind, placebo controlled	718 patients (>12 years old) with moderate to severe AA	Range of daily oral dosing for a 4-week loading phase/20-week maintenance phase/24-week extension phase: 200 mg/50 mg/50 mg 200 mg/30 mg/30 mg 50 mg/50 mg/50 mg 30 mg/30 mg/30 mg 10 mg/10 mg/10 mg placebo	The estimated rate achieving a SALT score ≦20 was 31% with 200/50/50 mg, 22% with 200/30/30 mg, 24% with 50/50/50 mg, 14.5% with 30/30/30 mg, and 2% with placebo.
Brepocitinib (JAK1/TYK2) [147]	Phase 2a, double blind	70 adult patients with moderate to severe AA	60 mg daily for 4 weeks, subsequently 30 mg daily for 20 weeks or placebo	64% of the patients receiving brepocitinib achieved SALT 30, and 34% achieved SALT 90 at 24 weeks.
Baricitinib (JAK1/2) [133]	Phase 3, double blind	1200 adult patients with severe AA	4 mg daily, 2 mg daily, or placebo	The estimated rate of the patients achieved SALT score of 20 or less at week 34 was 35.9–38.8% with 4 mg baricitinib, 19.4–22.8% with 2 mg baricitinib, and 3.3–6.2% with placebo.
CTP-543 (JAK1/2) [150]	Phase 2, randomized, dose-ranging trial	149 adult patients with moderate to severe AA	4 mg, 8 mg, or 12 mg twice daily or placebo for 24 weeks	The percentage of patients with ≥50% reduction of SALT score at week 24 was 58% with 12 mg CTP-543, 47% with 8 mg CTP-543, 21% with 4 mg CTP-543, and 9% with placebo.
Apremilast [174]	A double-blind, placebo-controlled single-center pilot study	30 adult patients with moderate to severe AA	30 mg daily or placebo for 24 weeks	The percentage of patients with ≥50% reduction of SALT score at week 24 was 8.3% with apremilast and 12.5% with placebo.

AA, alopecia areata; JAK, Janus kinase; SALT, Severity of Alopecia Tool.

### 3.3. Telogen Effluvium

#### 3.3.1. Etiopathogenesis

Telogen effluvium (TE) is a hair loss condition typically manifesting an acute and diffuse hair loss caused by internal or external insults [175]. Premature termination of the anagen (growing) phase of the hair cycle is the main pathomechanism of TE, which leads to excessive and diffuse loss of club hairs [176]. Various conditions, such as febrile or infectious diseases, childbirth, major surgy, chronic systemic diseases, emotional disturbance, or medications, can cause TE [176]. Three major types of TE, (1)~(3), have been proposed, based on the clinical time-course and the clearness in the association with causative events/conditions [177]. (1) An acute form of TE lasts less than 6 month and is typically with a sudden onset, while chronic forms of TE last longer [175,177]. The latter includes (2) chronic TE as an idiopathic subtype and (3) chronic diffuse telogen hair loss mostly due to systemic medical problems [175,177]. A chief complain of TE patients is usually the increase in hair shedding noticed during shampooing or brushing [177]. Prognosis of TE is usually favorable when triggers or causes are no longer persisting or already identified and resolved [177,178].

Probably because of its self-healing nature, TE does not attract great interest as a topic of clinicopathological investigation. Still, some updates can be made.

Five respective pathomechanisms, (1)~(5), have been proposed by Headington [179]. This categorization explains the major clinicopathological characteristics of TE well and is therefore widely accepted: (1) immediate anagen release—premature termination of the anagen phase and subsequent entry into the telogen phase (main pathophysiology explaining acute TE cause by triggering events and causes); (2) delayed anagen release—prolongation of the anagen phase with sudden termination (possibly accounts for postpartum hair loss); (3) short anagen—idiopathic shortening of the anagen phase (e.g., short anagen syndrome or etretinate-induced hair loss); (4) immediate telogen release—a consequence of synchronous entry into new anagen phase (can be seen after topical minoxidil application); and (5) delayed telogen release—rapid entry into the anagen phase after sustained telogen (seasonal hair shedding in mammals). Recently, Rebora proposed a simpler classification of TE [180]. In this classification, the term “teloptosis”, referring to the detachment of the hair shaft produced in the preceding hair cycle from the follicular epithelial sac, is featured [2]. Three pathomechanisms, (1)~(3), are included. Premature teloptosis corresponds to the abovementioned (1) “immediate telogen release”. Premature teloptosis may also include (2) “delayed anagen and telogen release” resulting from the synchronization of the hair cycles (e.g., postpartum TE or the effluvium of the newborn). (3) Premature entry to the telogen phase can correspond to “immediate anagen release” induced by various triggers. To what extent the addition of this new classification deepens our understanding of TE etiopathogenesis remains elusive. Still, the less-complicated categorization can be more easily applicable to daily practice.

Chronic TE is an idiopathic and unique form of TE that is typically seen in a middle-aged woman [177]. Hair shedding lasts longer for several years, with fluctuation [177]. Chronic TE patients typically claim extensive hair shedding, and telogen club hairs can easily be pulled out from the vertex and occipital areas; however, visual hair thinning is often minimal [177]. Bitemporal recession of hair can be observed [181]. The condition needs to be differentiated from chronic diffuse telogen hair loss and female pattern hair loss [177]. A recent study reported the association between the Cdx1 and TaqI polymorphism of the VDR gene and chronic TE, suggesting a possible genetic predisposition [182].

Febrile and/or infectious diseases are known TE triggers, and hair loss during the influenza epidemic has been described [183]. As described below in the section of COVID-19 and hair loss, the majority of COVID-19 hair loss has been reported to be TE due to cytokine storm, physical exhaustion, and lockdown stress [184]. More recently, Dengue fever has been reported as a possible trigger of acute TE [185].

#### 3.3.2. Treatment

Essentially, TE can be improved once causative factors are eliminated. Course observation waiting for spontaneous resolution and psychological support with assurance of hair regrowth represent the main approaches for managing TE [176,177,186]. Because of its distressing nature, more positive therapeutic approaches could be applied to chronic TE; however, options are still limited. As a careful consideration for advantage and disadvantage balance is indispensable for the use of oral minoxidil, topical minoxidil can be a possible option [184]. Levels of evidence for other treatment modalities, such as minerals, vitamins, and nutrition supplementation; the use of antioxidant and anti-inflammatory shampoo/lotion; photobiostimulation; and microneedling are still not high, highlighting the necessity of further evaluation [184].

### 3.4. COVID-19 Associated Hair Loss

Despite the fact that the incidence of “Long COVID” associated with the currently spreading omicron variant of SARS-COV-2 has been reported to be low when compared to that caused by the delta variants [187] and that hair loss seems to be less frequently discussed in the context of COVID-19 sequelae, hair loss after COVID 19 has still been imprinted into the public memory as a representative symptom [184]. The frequency of hair loss prior to the omicron variant surge has been reported to be 6–28.6% [184]. A systematic review and meta-analysis found that AGA, AA, and TE were associated with COVID-19 [20].

#### 3.4.1. Etiopathogenesis

An increase in the prevalence of AGA has been reported in COVID-19; however, AGA mostly precedes COVID-19, suggesting that AGA can be a risk factor rather than a sequela of COVID-19 [20]. Indeed, the incidence of hospitalization due to severe COVID-19 was higher in AGA-affected individuals (both in male and female) than that in those without AGA, suggesting that the presence of AGA could increase the chance of COVID-19 aggravation [20]. Androgen-mediated transmembrane serine protease 2 upregulation promoting SARS-CoV-2 virion entry has been speculated to be a possible mechanism underlying this observation [188], as implied by the results of clinical studies reporting the protective effects of androgen-deprivation therapies [189,190].

Because of its autoimmune-mediated pathophysiology and possible association with psychological stress due to quarantine or sociological restrictions as triggers, AA has been speculated to be caused or exacerbated by COVID-19 [191]. A recent nationwide cohort study from the Republic of Korea analyzing the National Health Insurance Service COVID-19 cohort database reported that the adjusted incidence ratio of AA development among COVID-19 patients was 0.78 (95% CI: 0.48–1.27) compared to controls when adjusted for age and gender and 0.60 (95% CI: 0.35–1.03), after the adjustment for all demographic variables [191]. In contrast, 95.1% of AA cases with a history of COVID-19 were found to have preexisting AA, and the post-COVID-19 relapse rate of AA was reported to be 42.5% [187]. These data suggested that COVID-19 could increase the risk of worsening of preexisting AA but might not be closely associated with a new onset. Possible associations with vaccines and the pathogenesis of autoimmune diseases have been discussed [192]. As COVID-19 vaccines elicit inflammatory responses, including the increase in the secretion of IFN and IL-6, which have been implicated in AA pathogenesis, their putative role of triggering AA has been a topic of global interest [192]. A recent case report and review of the scientific literature suggested that COVID-19 vaccines may induce AA in predisposed individuals, particularly in those with anti-thyroid peroxidase antibodies [192]. Still, further accumulation of the cases is mandated to draw a definitive conclusion.

**Figure 8:**
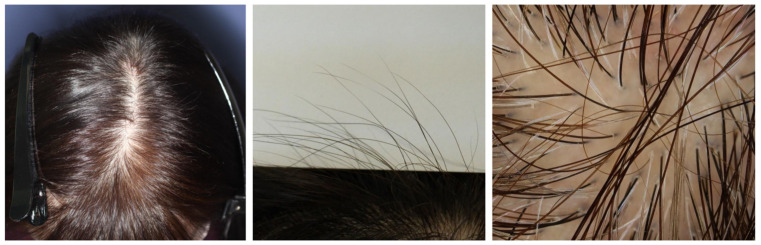
Clinical findings of hair loss two months after COVID-19. This case showed typical manifestation of telogen effluvium: diffuse hair loss (**left panel**); upright regrowing hairs, as highlighted by white background (**middle panel**); and trichoscopy (**right panel**).

Recent reviews of the scientific literature elucidated that TE represents the main pathomechanism of hair loss after COVID-19 (Figure 8) [20,184], and this was further supported by a recent histopathological analysis [193]. A survey of the scientific literature found that the median duration between COVID-19 and TE onset was 56.5 days, slightly shorter than that reported in classic acute TE [20]. As described above, the increase in inflammatory cytokines (e.g., IFN and IL-6) in COVID-19, together with other factors, such as genetic susceptibility, severity of COVID-19, and physiological stress, should play a role in casing TE [184].

#### 3.4.2. Treatment

As acute TE usually improves once causative events are cleared and TE predominates COVID-19-associated hair loss, the treatment of COVID-19 by itself represents a main therapeutic approach [184]. Topical minoxidil may enhance improvement when it is used in and after the recovery phase of COVID-19 [184].

## 4. Cicatricial Alopecia

### 4.1. Etiopathogenesis

#### 4.1.1. Lichen Planopilaris (LPP) and Frontal Fibrosing Alopecia (FFA)

LPP is a major inflammatory cicatricial alopecia, and FFA is considered its variant. Some overlapping features in the etiopathomechanism have been suggested for LPP and FFA [194,195]. CD8+ T-cell-dominated lymphocytic cell infiltration damaging HFs after the collapse of IP involving the bulge stem cell region has been thought to play a main role in both conditions [195]. Epidermal–mesenchymal transition (EMT), insufficiency in PPAR-γ-mediating signaling [195], and dysregulation of shared gene expressions [196] are potentially involved in the pathogenesis of both conditions. At the same time, characteristics distinguishing LPP and FFA have been suggested in recent studies. An immunohistopathological analysis by Harries et al. reported that lesional increase in M2 macrophages was apparent in LPP compared to FFA [195]. A recent genetic analysis revealed that LPP possesses significantly higher frequency of the HLA DRB1*11 and DQB1*03 [197]. In contrast, another GWAS study reported that four loci, including *CYP1B1* and HLA-B*07:02, are susceptible for FFA [198]. Indeed, familial FFA cases have been reported in previous articles suggesting the presence of genetic predisposition in this condition [199,200]. The contribution of environment factors in each pathogenesis can distinguish FFA from classical LPP. The use of skin care products is considered a potential causative factor for each pathogenesis. A recent meta-analysis demonstrated that sunscreen or moisturizer use is associated, respectively, with a 2.21- or 2.09-times higher risk of developing FFA [18]. How these putative factors contribute to FFA pathogenesis remains elusive; allergic contact dermatitis and lichenoid reactions, generation of reactive oxygen species, and hormone disruption have been suggested to be underlying pathomechanisms [18]. In a recent retrospective analysis of 168 LPP and FFA patients, androgen excess was identified in 31.5% of LPP, whereas androgen deficiency was identified in 32.1% of FFA, with statistical significance in both ratios [201], implying these two subgroups are distinctive in androgen dynamics.

These findings imply that LPP and FFA share the same pathomechanism as stem to a certain extent but are also influenced by distinct and diverse factors eventually resulting in respective clinical presentations. Thus, these two entities can be likened to “two distinct branches of the same tree” [16].

#### 4.1.2. Fibrosing Alopecia in a Pattern Distribution (FAPD)

**Figure 9:**
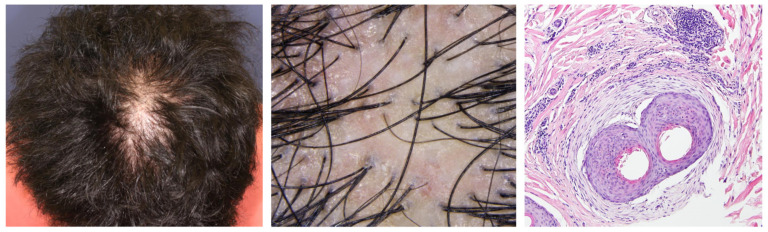
Representative clinical and histopathological findings of fibrosing alopecia in a pattern distribution. Hair loss with hair miniaturization on the parietal area mimicking androgenetic alopecia (**left panel**). Loss of follicular openings and perifollicular erythema were also detected on trichoscopy (**middle panel**), and histopathological examination showed perifollicular cell infiltration with concentric perifollicular lamellar fibrosis mimicking lichen planopilaris (**right panel**).

FAPD is a relatively rare entity demonstrating mixed characteristics of LPP and AGA (Figure 9) [5]. Its etiopathology is still ill understood; however, the inflammatory process preferentially attacking miniaturized HFs has been speculated to be a main pathomechanism [5]. According to a review of the literature covering previous articles, the average age at diagnosis was 55.1 for women and 46.5 for men [5]; however, a recent study from Japan reported that Japanese male patients tend to develop this condition far earlier, and also their hair loss pattern is distinctive, frequently involving the vertex area, with minimal recession of the frontal hairline, indicating that racial predisposition may contribute to its pathophysiology and clinical phenotypes [202].

#### 4.1.3. Central Centrifugal Cicatricial Alopecia (CCCA)

CCCA typically affects African American women, and thus, genetic background has been speculated to influence its pathogenesis [203,204]. Importantly, a recent genetic study revealed that mutations in *PADI3*, the gene encoding a protein essential to proper hair shaft formation, are associated with CCCA [203]. Intriguingly, some conditions/diseases have been reported in association with CCCA. An American population study revealed that the ratio of diabetes mellitus type 2 was significantly higher in those with CCCA [205]. A retrospective study adopting a medical record review reported an increased risk of uterine leiomyoma [206]; however, a microarray gene analysis study found no signs of upregulation in genes associated with fibroproliferative disorders in CCCA patients [207].

#### 4.1.4. Folliculitis Decalvans (FD)

FD was categorized into the “neutrophilic” type of PCA in the classification proposed in 2003 by North American Hair Research Society (NAHRS), distinguishing this disease from LPP and CCCA, which were both categorized into the “lymphocytic” type [208]. However, recent reports described the cases manifesting clinicopathological features of both FD and LPP, and the term “FD/LPP phenotypic spectrum” (FDLPPPS) has been proposed to describe this condition [209,210]. Based on the observation of eight FDLPPPS cases in which FD proceeded LPP, Yip et al. speculated that dysbiosis in the skin microbiome may elicit damaging stress signals and propagate inflammatory response in the microenvironment, with resultant IP collapse and exposure of follicular self-antigens as a possible explanation for the pathomechanism [209]. They also hypothesized that forms of FDLPPPS corresponded to the phenotypic manifestation of a final common pathway seen in all scarring alopecia before the HF is ultimately destroyed [209]. Matard et al. supported the hypothesis of Yip and colleagues based on their experience and the literature data, warning that this condition needs to be differentiated from real LPP from the therapeutic point of view [211].

#### 4.1.5. Other Types of Cicatricial Alopecia

The associations between other miscellaneous primary cicatricial alopecia (PCA) and comorbidities are updated. Follicular (acne) keloidalis (FK) is one form of PCA that is categorized in the “mixed (lymphocytic and neutrophilic)” group [208]. Shavit et al. reported the increased risk of gout in FK based on the result of a population-based study [212]. As the statistical significance was lost after the adjustment for metabolic comorbidities, the authors speculated that the elevated risk of gout in FK is mainly explained by the predominance of metabolic comorbidities in FK [212]. Another study demonstrated that widespread FK can be seen in the setting of cutis verticis gyrata [213], a condition with thickening of the scalp skin with excessive folds. Dissecting cellulitis (DC) (perifolliculitis abscedens et suffodiens) is an independent form of PCA [208]. Zagelbaum Ward et al. reported a case of DC with peripheral and axial spondyloarthritis with a review of the literature which identified 12 DC patients with associated spondyloarthropathy [214]. The association between seronegative peripheral and/or axial spondyloarthritis in patients with hidradenitis suppurativa and acne conglobate, two conditions forming “follicular occlusion triad” with DC, is well established; and thus, the authors’ report further supports that the conditions in this triad share a predilection toward certain immunological conditions [214].

### 4.2. Treatment

As each of the PCAs share the same pathophysiology in which inflammation involving the bulge stem cell area of HF with resultant permanent loss of HFs, anti-inflammatory agents have been conventionally applied to all types of alopecia. Therapeutic approaches include topical, intralesional, and systemic corticosteroid combined with immunosuppressants or immunomodulating modalities such as hydroxychloroquine, mycophenolate, cyclosporine, methotrexate, and retinoids. For neutrophilic conditions, antibacterial agents, including doxycycline, minocycline, azithromycin, clindamycin, and rifampicin, are also considered [215,216,217]. Recently reported therapeutic options for PCA are listed below.

#### 4.2.1. PPAR-γ Agonist

The dysfunction of PPAR-γ and dysregulation of PPAR-γ-stimulating lipid mediators have been postulated to be critical factors in the pathogenesis of LPP [194]. Based on this hypothesis, PPAR-γ agonists have been attracting interest as potential therapeutic medication for LPP. Some studies reported that oral pioglitazone was successful in controlling symptoms, inflammation, or disease progression [218,219,220], while other studies reported negative results with regards to continuity of improvement or curative efficacy [221]. Most recently, a randomized clinical trial was conducted to compare the efficacy of oral pioglitazone with that of topical clobetasol. Both treatments demonstrated a significant decrease in LPPAI (aforementioned activity index) without significant difference between two groups [222]. As to the safety profile, three out of 20 in the clobetasol group developed folliculitis, whereas two out of 20 in the pioglitazone group developed mild headaches. The outcome indicated that pioglitazone can be considered as a treatment option for LPP, but its efficacy is comparable to that of topical clobetasol [222].

#### 4.2.2. JAK Inhibitors

Several reports suggested the efficacy of JAK inhibitors for LPP/FFA. Yang et al. retrospectively reviewed 10 cases with recalcitrant LPP/FFA treated by oral tofacitinib 10–15 mg/day for 2–19 months [223]. Eight out of ten patients demonstrated clinical improvement, and a reduction in LPPAI score, ranging from 30 to 94%, in improved cases was observed [223]. Another retrospective case series reported the clinical outcome of nine LPP/FFA treated by topical or oral tofacitinib, in which all six patients on oral tofacitinib demonstrated improvement in LPPAI scores [224]. A most recent retrospective review published in 2022 reported that five out of seven classical LPPs and three out of the five FFA patients demonstrated a reduction in their LPPAI score achieved by oral baricitinib, a JAK1/2 inhibitor [225]. No severe adverse event was reported [223,224,225]. These outcomes may highlight the therapeutic potential of JAK inhibitors for LPP/FFA. However, the true efficacy of them in LPP/FFA still remains unclear, as they were used with other therapeutic modalities in most cases [223,224,225]. Studies with a higher evidence level are needed to evaluate the bona fide efficacy of JAK inhibitors. The exact influence of JAK inhibitors on the pathogenesis of LPP/FFA has not been elucidated; however, a recent research study demonstrated that IFN-γ enhanced cell-mediated cytotoxicity against keratinocytes via JAK2/STAT1 in lichen planus, supporting the idea that JAK inhibitors hold promise as therapeutic agents in LPP/FFA [226].

#### 4.2.3. Biologics

The benefit of TNF-α inhibitors in treating neutrophilic PCA resistant to initial standard therapies has been suggested [215,216,227]. A systematic review of 57 articles describing the outcomes of treatment switch due to insufficient effectiveness of conventional therapeutic approaches in refractory DC reported favorable responses to adalimumab and infliximab [215]. A recent multicenter study including 26 DC patients treated by TNF-α inhibitors reported that the mean physician global assessment score decreased by 56% with the decrease in the number of inflammatory nodules (−70%), the number of abscesses (−89%) after a mean treatment duration of 19 ± 21 months [227]. Moreover, a recent case series reported 23 FD cases refractory to conventional treatments and thus treated by adalimumab alone demonstrated clinically meaningful responses [216]. Considering that TNF-α is a main player in neutrophilic dermatoses and that a higher level of evidence exists for the efficacy of TNF-α inhibitors in some of those conditions [228], the use of these modalities may benefit neutrophilic PCAs represented by DC and FD.

## 5. Conclusions

Major advances have been made in the understanding, diagnosis, and management of hair diseases. However, hair loss diseases are a complex entity with still undiagnosed/undefined conditions. Despite recent progress in therapeutic approaches, the effectiveness of newly invented medications, for instance, that of JAK inhibitors for severe AA, needs to be improved. A further dissection of pathomechanism and a new drug discovery or invention of novel diagnostic and therapeutic modalities, in combination with the accumulation of evidence-based data, are necessary to fully overcome this potentially intractable disease complex.

## Figures and Tables

**Table 2:** Representative trichoscopic findings, definitions, and suggestive diseases.

Findings	Definitions	Suggestive Diseases *
**Changes in hair shafts**		
Black dot	Fractured hair with dotted shape seen in hair ostia	AA
Broken hair	Short hair with dystrophic (or broken) peripheral ending	AA
Coudability hair (tapered hair)	Terminal hair with tapered proximal shaft	AA
Exclamation-mark hair (tapering hair)	Short and fractured hair with gradual thinning of proximal shaft	AA
Pohl-Pinkus constriction	Irregular narrowing of hair shaft	AA
Short vellus hair	Thin and hypopigmented short hair	AA (recovering phase)
Upright regrowing hair	Short regrowing hair with straight-up position and tapered distal ending	AA (recovering phase)
**Changes in hair follicles**		
Absence of follicular openings	Disappearance of follicular openings	CA
Follicular keratotic plugging	Thick keratotic material filling follicular ostia	CA (DLE)
Follicular red dot	Erythematous polycyclic structure observed in and around follicular openings	CA (DLE)
White dots	Whitish fibrotic dotted area corresponding to disappeared follicular openings	CA (LPP)
Yellow dots	Yellowish keratotic material and/or sebum filling follicular ostia	AA, AGA/FPHL
**Changes in perifollicular area**		
Blue-gray dot	Blue-to-grey-pigmented dot annularly distributed or randomly scattered around hair shaft	CA (LPP)
Follicular pustule	Pustule corresponding to hair follicle	CA (FD and DC)
Perifollicular erythema	Erythema observed around hair shafts	CA (LPP)
Perifollicular scale	Scale observed around hair shafts	CA (LPP)
Perifollicular whitish halo	Fibrotic whitish halo around hair shaft	CA
**Changes on scalp**		
Pinpoint white dot	Small (<0.3 mm) white dot seen in interfollicular space	CCCA **
White patch	Non-structured and hypopigmented fibrotic area	CA
**Changes in hair distribution**		
Hair diameter diversity	Distribution of randomly thinned hairs within the same field	AGA/FPHL
Focal atrichia	Small area lacking hairs	AGA/FPHL
Tufted hairs	Multiple terminal hairs from one hair ostia	CA (FD)

AA, alopecia areata; AGA, androgenetic alopecia; CA, cicatricial alopecia; DC, dissecting cellulitis; DLE, discoid lupus erythematosus; FD, folliculitis decalvans; FPHL, female pattern hair loss; LPP, lichen planopilaris. * Parentheses include particularly suggestive diseases or conditions. ** Pinpoint white dots are found in normal dark scalp but can be irregularly distributed in CCCA.

## Data Availability

The raw data of this article will be made available by the authors, without undue reservation.

## References

[1] Fukuyama M., Ito T., Ohyama M. (2022). Alopecia areata: Current understanding of the pathophysiology and update on therapeutic approaches, featuring the Japanese Dermatological Association guidelines. J. Dermatol..

[2] Chien Yin G.O., Siong-See J.L., Wang E.C.E. (2021). Telogen Effluvium—A review of the science and current obstacles. J Dermatol Sci.

[3] Uchiyama M. (2022). Primary cicatricial alopecia: Recent advances in evaluation and diagnosis based on trichoscopic and histopathological observation, including overlapping and specific features. J. Dermatol..

[4] Olsen E.A., Harries M., Tosti A., Bergfeld W., Blume-Peytavi U., Callender V., Chasapi V., Correia O., Cotsarelis G., Dhurat R. (2021). Guidelines for clinical trials of frontal fibrosing alopecia: Consensus recommendations from the International FFA Cooperative Group (IFFACG). Br. J. Dermatol..

[5] Griggs J., Trüeb R.M., Gavazzoni Dias M.F.R., Hordinsky M., Tosti A. (2021). Fibrosing alopecia in a pattern distribution. J. Am. Acad. Dermatol..

[6] Lee W.S., Ro B.I., Hong S.P., Bak H., Sim W.Y., Kim D.W., Park J.K., Ihm C.W., Eun H.C., Kwon O.S. (2007). A new classification of pattern hair loss that is universal for men and women: Basic and specific (BASP) classification. J. Am. Acad. Dermatol..

[7] Harries M., Tosti A., Bergfeld W., Blume-Peytavi U., Shapiro J., Lutz G., Messenger A., Sinclair R., Paus R. (2016). Towards a consensus on how to diagnose and quantify female pattern hair loss—The ’Female Pattern Hair Loss Severity Index (FPHL-SI)’. J. Eur. Acad. Dermatol. Venereol..

[8] Kasprzak M., Sicińska J., Sinclair R. (2019). The Trichoscopy Derived Sinclair Scale: Enhancing visual assessment through quantitative trichoscopy. Australas J. Dermatol..

[9] Kinoshita-Ise M., Sachdeva M. (2022). Update on trichoscopy: Integration of the terminology by systematic approach and a proposal of a diagnostic flowchart. J. Dermatol..

[10] McDonald K.A., Shelley A.J., Maliyar K., Abdalla T., Beach R.A., Beecker J. (2021). Hair pull test: A clinical update for patients with Asian- and Afro-textured hair. J. Am. Acad. Dermatol..

[11] McDonald K.A., Shelley A.J., Colantonio S., Beecker J. (2017). Hair pull test: Evidence-based update and revision of guidelines. J. Am. Acad. Dermatol..

[12] Kinoshita-Ise M., Ohyama M., Ramjist J.M., Foster F.S., Yang V.X.D., Sachdeva M., Sade S., Shear N.H. (2021). Ultra high-frequency ultrasound with seventy-MHz transducer in hair disorders: Development of a novel noninvasive diagnostic methodology. J. Dermatol. Sci..

[13] Rajabi-Estarabadi A., Vasquez-Herrera N.E., Martinez-Velasco M.A., Tsatalis J., Verne S.H., Nouri K., Tosti A. (2020). Optical coherence tomography in diagnosis of inflammatory scalp disorders. J. Eur. Acad. Dermatol. Venereol..

[14] Ohyama M. (2007). Hair follicle bulge: A fascinating reservoir of epithelial stem cells. J. Dermatol. Sci..

[15] Lavker R.M., Sun T.T., Oshima H., Barrandon Y., Akiyama M., Ferraris C., Chevalier G., Favier B., Jahoda C.A., Dhouailly D. (2003). Hair follicle stem cells. J. Investig. Derm. Symp. Proc..

[16] Harries M.J., Jimenez F., Izeta A., Hardman J., Panicker S.P., Poblet E., Paus R. (2018). Lichen Planopilaris and Frontal Fibrosing Alopecia as Model Epithelial Stem Cell Diseases. Trends Mol. Med..

[17] Redler S., Brockschmidt F.F., Tazi-Ahnini R., Drichel D., Birch M.P., Dobson K., Giehl K.A., Herms S., Refke M., Kluck N. (2012). Investigation of the male pattern baldness major genetic susceptibility loci AR/EDA2R and 20p11 in female pattern hair loss. Br. J. Dermatol..

[18] Maghfour J., Ceresnie M., Olson J., Lim H.W. (2022). The association between frontal fibrosing alopecia, sunscreen, and moisturizers: A systematic review and meta-analysis. J. Am. Acad. Dermatol..

[19] Betz R.C., Petukhova L., Ripke S., Huang H., Menelaou A., Redler S., Becker T., Heilmann S., Yamany T., Duvic M. (2015). Genome-wide meta-analysis in alopecia areata resolves HLA associations and reveals two new susceptibility loci. Nat. Commun..

[20] Nguyen B., Tosti A. (2022). Alopecia in patients with COVID-19: A systematic review and meta-analysis. JAAD Int..

[21] Ladizinski B., Bazakas A., Selim M.A., Olsen E.A. (2013). Frontal fibrosing alopecia: A retrospective review of 19 patients seen at Duke University. J. Am. Acad. Dermatol..

[22] Zinkernagel M.S., Trüeb R.M. (2000). Fibrosing alopecia in a pattern distribution: Patterned lichen planopilaris or androgenetic alopecia with a lichenoid tissue reaction pattern?. Arch. Dermatol..

[23] Olsen E.A., Hordinsky M.K., Price V.H., Roberts J.L., Shapiro J., Canfield D., Duvic M., King L.E., McMichael A.J., Randall V.A. (2004). Alopecia areata investigational assessment guidelines--Part II. National Alopecia Areata Foundation. J. Am. Acad. Dermatol..

[24] Olsen E.A., Canfield D. (2016). SALT II: A new take on the Severity of Alopecia Tool (SALT) for determining percentage scalp hair loss. J. Am. Acad. Dermatol..

[25] King B.A., Mesinkovska N.A., Craiglow B., Kindred C., Ko J., McMichael A., Shapiro J., Goh C., Mirmirani P., Tosti A. (2022). Development of the alopecia areata scale for clinical use: Results of an academic-industry collaborative effort. J. Am. Acad. Dermatol..

[26] Lesko S.M., Rosenberg L., Shapiro S. (1993). A case-control study of baldness in relation to myocardial infarction in men. JAMA.

[27] Ludwig E. (1977). Classification of the types of androgenetic alopecia (common baldness) occurring in the female sex. Br. J. Dermatol..

[28] Olsen E.A. (1999). The midline part: An important physical clue to the clinical diagnosis of androgenetic alopecia in women. J. Am. Acad. Dermatol..

[29] Biondo S., Goble D., Sinclair R. (2004). Women who present with female pattern hair loss tend to underestimate the severity of their hair loss. Br. J. Dermatol..

[30] Sinclair R., Wewerinke M., Jolley D. (2005). Treatment of female pattern hair loss with oral antiandrogens. Br. J. Dermatol..

[31] Tajima M., Hamada C., Arai T., Miyazawa M., Shibata R., Ishino A. (2007). Characteristic features of Japanese women’s hair with aging and with progressing hair loss. J. Dermatol. Sci..

[32] Kaneko A., Kaneko T. (2018). A New Classification of Early Female Pattern Hair Loss. Int. J. Trichology.

[33] Lee W.S., Lee H.J. (2012). Characteristics of androgenetic alopecia in asian. Ann. Dermatol..

[34] Chiang C., Sah D., Cho B.K., Ochoa B.E., Price V.H. (2010). Hydroxychloroquine and lichen planopilaris: Efficacy and introduction of Lichen Planopilaris Activity Index scoring system. J. Am. Acad. Dermatol..

[35] Holmes S., Ryan T., Young D., Harries M. (2016). Frontal Fibrosing Alopecia Severity Index (FFASI): A validated scoring system for assessing frontal fibrosing alopecia. Br. J. Dermatol..

[36] Saceda-Corralo D., Moreno-Arrones Ó.M., Fonda-Pascual P., Pindado-Ortega C., Buendía-Castaño D., Alegre-Sánchez A., Segurado-Miravalles G., Rodrigues-Barata A.R., Jaén-Olasolo P., Vaño-Galván S. (2018). Development and validation of the Frontal Fibrosing Alopecia Severity Score. J. Am. Acad. Dermatol..

[37] Dhurat R., Saraogi P. (2009). Hair evaluation methods: Merits and demerits. Int. J. Trichology.

[38] Mubki T., Rudnicka L., Olszewska M., Shapiro J. (2014). Evaluation and diagnosis of the hair loss patient: Part I. History and clinical examination. J. Am. Acad. Dermatol..

[39] Daly T., Daly K. (2018). Telogen Effluvium With Dysesthesia (TED) Has Lower B12 Levels and May Respond to B12 Supplementation. J. Drugs Dermatol..

[40] Blume-Peytavi U., Blumeyer A., Tosti A., Finner A., Marmol V., Trakatelli M., Reygagne P., Messenger A. (2011). S1 guideline for diagnostic evaluation in androgenetic alopecia in men, women and adolescents. Br. J. Dermatol..

[41] Conic R.R.Z., Piliang M., Bergfeld W., Atanaskova-Mesinkovska N. (2021). Vitamin D status in scarring and nonscarring alopecia. J. Am. Acad. Dermatol..

[42] Nosewicz J., Spaccarelli N., Roberts K.M., Hart P.A., Kaffenberger J.A., Trinidad J.C., Kaffenberger B.H. (2022). The epidemiology, impact, and diagnosis of micronutrient nutritional dermatoses part 1: Zinc, selenium, copper, vitamin A, and vitamin C. J. Am. Acad. Dermatol..

[43] Nosewicz J., Spaccarelli N., Roberts K.M., Hart P.A., Kaffenberger J.A., Trinidad J.C., Kaffenberger B.H. (2022). The epidemiology, impact, and diagnosis of micronutrient nutritional dermatoses. Part 2: B-complex vitamins. J. Am. Acad. Dermatol..

[44] Shim W.H., Jwa S.W., Song M., Kim H.S., Ko H.C., Kim B.S., Kim M.B. (2014). Dermoscopic approach to a small round to oval hairless patch on the scalp. Ann. Dermatol..

[45] Inui S. (2011). Trichoscopy for common hair loss diseases: Algorithmic method for diagnosis. J. Dermatol..

[46] Jain N., Doshi B., Khopkar U. (2013). Trichoscopy in alopecias: Diagnosis simplified. Int. J. Trichology.

[47] Waśkiel A., Rakowska A., Sikora M., Olszewska M., Rudnicka L. (2018). Trichoscopy of alopecia areata: An update. J. Dermatol..

[48] Waśkiel-Burnat A., Rakowska A., Sikora M., Ciechanowicz P., Olszewska M., Rudnicka L. (2020). Trichoscopy of Tinea Capitis: A Systematic Review. Dermatol. Ther..

[49] Mathur M., Acharya P. (2020). Trichoscopy of primary cicatricial alopecias: An updated review. J. Eur. Acad. Dermatol. Venereol..

[50] Żychowska M., Żychowska M. (2021). Dermoscopy of discoid lupus erythematosus—A systematic review of the literature. Int. J. Dermatol..

[51] Kaczorowska A., Rudnicka L., Stefanato C.M., Waskiel-Burnat A., Warszawik-Hendzel O., Olszewska M., Rakowska A. (2021). Diagnostic Accuracy of Trichoscopy in Trichotillomania: A Systematic Review. Acta Derm. Venereol..

[52] Almuhanna N., Wortsman X., Wohlmuth-Wieser I., Kinoshita-Ise M., Alhusayen R. (2021). Overview of Ultrasound Imaging Applications in Dermatology [Formula: See text]. J. Cutan. Med. Surg..

[53] Vazquez-Herrera N.E., Eber A.E., Martinez-Velasco M.A., Perper M., Cervantes J., Verne S.H., Magno R.J., Nouri K., Tosti A. (2018). Optical coherence tomography for the investigation of frontal fibrosing alopecia. J. Eur. Acad. Dermatol. Venereol..

[54] Ekelem C., Feil N., Csuka E., Juhasz M., Lin J., Choi F., Asghari A., Heydarlou D., Mesinkovska N.A. (2021). Optical Coherence Tomography in the Evaluation of the Scalp and Hair: Common Features and Clinical Utility. Lasers Surg. Med..

[55] Mai W., Sun Y., Liu X., Lin D., Lu D. (2019). Characteristic findings by phototrichogram in southern Chinese women with Female pattern hair loss. Skin Res. Technol..

[56] Rossi A., Magri F., Caro G., Michelini S., Di Fraia M., Fortuna M.C., Pellacani G., Carlesimo M. (2021). Fluorescence advanced videodermoscopy: A new method of hairs and scalp evaluation. Comparison with trichoscopy. J. Eur. Acad. Dermatol. Venereol..

[57] Napolitano M., Cantelli M., Potestio L., Ocampo-Garza S.S., Vastarella M., Nappa P., Scalvenzi M., Fabbrocini G., Patruno C. (2022). Clinical, trichoscopic and in vivo reflectance confocal microscopy evaluation of alopecia areata in atopic dermatitis patients treated with dupilumab. J. Eur. Acad. Dermatol. Venereol..

[58] Kurzeja M., Czuwara J., Walecka I., Olszewska M., Rudnicka L. (2021). Features of classic lichen planopilaris and frontal fibrosing alopecia in reflectance confocal microscopy: A preliminary study. Skin Res. Technol..

[59] Frishberg D.P., Sperling L.C., Guthrie V.M. (1996). Transverse scalp sections: A proposed method for laboratory processing. J. Am. Acad. Dermatol..

[60] Garcia C., Poletti E. (2007). Scalp biopsy specimens: Transverse vs. vertical sections. Arch. Dermatol..

[61] Ko J.H., Huang Y.H., Kuo T.T. (2012). Hair counts from normal scalp biopsy in Taiwan. Dermatol. Surg..

[62] Lee H.J., Ha S.J., Lee J.H., Kim J.W., Kim H.O., Whiting D.A. (2002). Hair counts from scalp biopsy specimens in Asians. J. Am. Acad. Dermatol..

[63] Aslani F.S., Dastgheib L., Banihashemi B.M. (2009). Hair counts in scalp biopsy of males and females with androgenetic alopecia compared with normal subjects. J. Cutan. Pathol..

[64] Sperling L.C. (1999). Hair density in African Americans. Arch. Dermatol..

[65] Whiting D.A. (1993). Diagnostic and predictive value of horizontal sections of scalp biopsy specimens in male pattern androgenetic alopecia. J. Am. Acad. Dermatol..

[66] Manabe M., Tsuboi R., Itami S., Osada S.I., Amoh Y., Ito T., Inui S., Ueki R., Ohyama M., Kurata S. (2018). Guidelines for the diagnosis and treatment of male-pattern and female-pattern hair loss, 2017 version. J. Dermatol..

[67] Rebora A. (2004). Pathogenesis of androgenetic alopecia. J. Am. Acad. Dermatol..

[68] Garza L.A., Liu Y., Yang Z., Alagesan B., Lawson J.A., Norberg S.M., Loy D.E., Zhao T., Blatt H.B., Stanton D.C. (2012). Prostaglandin D2 inhibits hair growth and is elevated in bald scalp of men with androgenetic alopecia. Sci. Transl. Med..

[69] Leirós G.J., Attorresi A.I., Balañá M.E. (2012). Hair follicle stem cell differentiation is inhibited through cross-talk between Wnt/β-catenin and androgen signalling in dermal papilla cells from patients with androgenetic alopecia. Br. J. Dermatol..

[70] Chew E.G.Y., Lim T.C., Leong M.F., Liu X., Sia Y.Y., Leong S.T., Yan-Jiang B.C., Stoecklin C., Borhan R., Heilmann-Heimbach S. (2022). Observations that suggest a contribution of altered dermal papilla mitochondrial function to androgenetic alopecia. Exp. Dermatol..

[71] Trüeb R.M. (2015). The impact of oxidative stress on hair. Int. J. Cosmet. Sci..

[72] Ho B.S., Vaz C., Ramasamy S., Chew E.G.Y., Mohamed J.S., Jaffar H., Hillmer A., Tanavde V., Bigliardi-Qi M., Bigliardi P.L. (2019). Progressive expression of PPARGC1α is associated with hair miniaturization in androgenetic alopecia. Sci. Rep..

[73] Liu W., Li K., Wang G., Yang L., Qu Q., Fan Z., Sun Y., Huang J., Miao Y., Hu Z. (2021). Impairment of autophagy may be associated with follicular miniaturization in androgenetic alopecia by inducing premature catagen. J. Dermatol..

[74] Su L.H., Chen T.H. (2010). Association of androgenetic alopecia with metabolic syndrome in men: A community-based survey. Br. J. Dermatol..

[75] Bhat Y.J., Saqib N.U., Latif I., Hassan I. (2020). Female Pattern Hair Loss-An Update. Indian Derm. Online J..

[76] Fabbrocini G., Cantelli M., Masarà A., Annunziata M.C., Marasca C., Cacciapuoti S. (2018). Female pattern hair loss: A clinical, pathophysiologic, and therapeutic review. Int. J. Womens Dermatol..

[77] Paik J.H., Yoon J.B., Sim W.Y., Kim B.S., Kim N.I. (2001). The prevalence and types of androgenetic alopecia in Korean men and women. Br. J. Dermatol..

[78] Ohn J., Son H.Y., Yu D.A., Kim M.S., Kwon S., Park W.S., Kim J.I., Kwon O. (2022). Early onset female pattern hair loss: A case-control study for analyzing clinical features and genetic variants. J. Dermatol. Sci..

[79] Olsen E.A., Messenger A.G., Shapiro J., Bergfeld W.F., Hordinsky M.K., Roberts J.L., Stough D., Washenik K., Whiting D.A. (2005). Evaluation and treatment of male and female pattern hair loss. J. Am. Acad. Dermatol..

[80] Endo Y., Takahashi M., Obayashi Y., Serizawa T., Murakoshi M., Ohyama M. (2017). The ovariectomized mouse simulates the pathophysiology of postmenopausal female pattern hair loss. J. Dermatol. Sci..

[81] Endo Y., Obayashi Y., Ono T., Serizawa T., Murakoshi M., Ohyama M. (2018). Reversal of the hair loss phenotype by modulating the estradiol-ANGPT2 axis in the mouse model of female pattern hair loss. J. Dermatol. Sci..

[82] Mahé Y.F., Michelet J.F., Billoni N., Jarrousse F., Buan B., Commo S., Saint-Léger D., Bernard B.A. (2000). Androgenetic alopecia and microinflammation. Int. J. Dermatol..

[83] Ho B.S., Ho E.X.P., Chu C.W., Ramasamy S., Bigliardi-Qi M., de Sessions P.F., Bigliardi P.L. (2019). Microbiome in the hair follicle of androgenetic alopecia patients. PLoS ONE.

[84] Özkoca D., Aşkın Ö., Engin B. (2022). The comparison of demographics and comorbidities of female pattern hair loss according to the clinical subtype and stage. J. Am. Acad. Dermatol..

[85] Dominguez-Santas M., Diaz-Guimaraens B., Saceda-Corralo D., Hermosa-Gelbard A., Muñoz-Moreno Arrones O., Pindado-Ortega C., Fernandez-Nieto D., Jimenez-Cauhe J., Ortega-Quijano D., Suarez-Valle A. (2022). The state-of-the-art in the management of androgenetic alopecia: A review of new therapies and treatment algorithms. JEADV Clin. Pract..

[86] Gupta A.K., Venkataraman M., Talukder M., Bamimore M.A. (2022). Relative Efficacy of Minoxidil and the 5-α Reductase Inhibitors in Androgenetic Alopecia Treatment of Male Patients: A Network Meta-analysis. JAMA Dermatol..

[87] Choi G.S., Sim W.Y., Kang H., Huh C.H., Lee Y.W., Shantakumar S., Ho Y.F., Oh E.J., Duh M.S., Cheng W.Y. (2022). Long-Term Effectiveness and Safety of Dutasteride versus Finasteride in Patients with Male Androgenic Alopecia in South Korea: A Multicentre Chart Review Study. Ann. Dermatol..

[88] Shum K.W., Cullen D.R., Messenger A.G. (2002). Hair loss in women with hyperandrogenism: Four cases responding to finasteride. J. Am. Acad. Dermatol..

[89] Won Y.Y., Lew B.L., Sim W.Y. (2018). Clinical efficacy of oral administration of finasteride at a dose of 2.5 mg/day in women with female pattern hair loss. Dermatol. Ther..

[90] Olsen E.A., Hordinsky M., Whiting D., Stough D., Hobbs S., Ellis M.L., Wilson T., Rittmaster R.S. (2006). The importance of dual 5alpha-reductase inhibition in the treatment of male pattern hair loss: Results of a randomized placebo-controlled study of dutasteride versus finasteride. J. Am. Acad. Dermatol..

[91] Sung C.T., Juhasz M.L., Choi F.D., Mesinkovska N.A. (2019). The Efficacy of Topical Minoxidil for Non-Scarring Alopecia: A Systematic Review. J. Drugs Dermatol..

[92] Caserini M., Radicioni M., Leuratti C., Terragni E., Iorizzo M., Palmieri R. (2016). Effects of a novel finasteride 0.25% topical solution on scalp and serum dihydrotestosterone in healthy men with androgenetic alopecia. Int. J. Clin. Pharmacol. Ther..

[93] Blume-Peytavi U., Hillmann K., Dietz E., Canfield D., Garcia Bartels N. (2011). A randomized, single-blind trial of 5% minoxidil foam once daily versus 2% minoxidil solution twice daily in the treatment of androgenetic alopecia in women. J. Am. Acad. Dermatol..

[94] Lachgar S., Charveron M., Gall Y., Bonafe J.L. (1998). Minoxidil upregulates the expression of vascular endothelial growth factor in human hair dermal papilla cells. Br. J. Dermatol..

[95] Pietrauszka K., Bergler-Czop B. (2022). Sulfotransferase SULT1A1 activity in hair follicle, a prognostic marker of response to the minoxidil treatment in patients with androgenetic alopecia: A review. Postępy Dermatol. Alergol..

[96] Chitalia J., Dhurat R., Goren A., McCoy J., Kovacevic M., Situm M., Naccarato T., Lotti T. (2018). Characterization of follicular minoxidil sulfotransferase activity in a cohort of pattern hair loss patients from the Indian Subcontinent. Dermatol. Ther..

[97] Randolph M., Tosti A. (2021). Oral minoxidil treatment for hair loss: A review of efficacy and safety. J. Am. Acad. Dermatol..

[98] Sinclair R.D. (2018). Female pattern hair loss: A pilot study investigating combination therapy with low-dose oral minoxidil and spironolactone. Int. J. Dermatol..

[99] Shapiro J., Ho A., Sukhdeo K., Yin L., Lo Sicco K. (2020). Evaluation of platelet-rich plasma as a treatment for androgenetic alopecia: A randomized controlled trial. J. Am. Acad. Dermatol..

[100] Evans A.G., Mwangi J.M., Pope R.W., Ivanic M.G., Botros M.A., Glassman G.E., Pearce F.B., Kassis S. (2022). Platelet-rich plasma as a therapy for androgenic alopecia: A systematic review and meta-analysis. J. Dermatol. Treat..

[101] Elena E.P., Irina O.S. (2022). Combination therapy with platelet-rich plasma and minoxidil leads to better clinical results than monotherapy with these methods in men with androgenetic alopecia. Int. J. Trichology.

[102] Alves R., Grimalt R. (2018). Platelet-Rich Plasma in Combination With 5% Minoxidil Topical Solution and 1 mg Oral Finasteride for the Treatment of Androgenetic Alopecia: A Randomized Placebo-Controlled, Double-Blind, Half-Head Study. Dermatol. Surg..

[103] Gabbard R.D., Hoopes R.R., Kemp M.G. (2020). Spironolactone and XPB: An Old Drug with a New Molecular Target. Biomolecules.

[104] Aguilar Medina D.A., Cazarin J., Magana M. (2022). Spironolactone in dermatology. Dermatol. Ther..

[105] van Zuuren E.J., Fedorowicz Z., Schoones J. (2016). Interventions for female pattern hair loss. Cochrane Database Syst. Rev..

[106] Burns L.J., De Souza B., Flynn E., Hagigeorges D., Senna M.M. (2020). Spironolactone for treatment of female pattern hair loss. J. Am. Acad. Dermatol..

[107] Abdel-Raouf H., Aly U.F., Medhat W., Ahmed S.S., Abdel-Aziz R.T.A. (2021). A novel topical combination of minoxidil and spironolactone for androgenetic alopecia: Clinical, histopathological, and physicochemical study. Dermatol. Ther..

[108] Ammar A.M., Elshahid A.R., Abdel-Dayem H.A., Mohamed A.A., Elsaie M.L. (2022). Dermoscopic evaluation of the efficacy of combination of topical spironolactone 5% and minoxidil 5% solutions in the treatment of androgenetic alopecia: A cross sectional-comparative study. J. Cosmet. Dermatol..

[109] Wei C., Bovonratwet P., Gu A., Moawad G., Silverberg J.I., Friedman A.J. (2020). Spironolactone use does not increase the risk of female breast cancer recurrence: A retrospective analysis. J. Am. Acad. Dermatol..

[110] Vexiau P., Chaspoux C., Boudou P., Fiet J., Jouanique C., Hardy N., Reygagne P. (2002). Effects of minoxidil 2% vs. cyproterone acetate treatment on female androgenetic alopecia: A controlled, 12-month randomized trial. Br. J. Dermatol..

[111] Azarchi S., Bienenfeld A., Lo Sicco K., Marchbein S., Shapiro J., Nagler A.R. (2019). Androgens in women: Hormone-modulating therapies for skin disease. J. Am. Acad. Dermatol..

[112] Rossi A., Magri F., D’Arino A., Pigliacelli F., Muscianese M., Leoncini P., Caro G., Federico A., Fortuna M.C., Carlesimo M. (2020). Efficacy of Topical Finasteride 0.5% vs 17alpha-Estradiol 0.05% in the Treatment of Postmenopausal Female Pattern Hair Loss: A Retrospective, Single-Blind Study of 119 Patients. Dermatol. Pract. Concept..

[113] Egger A., Resnik S.R., Aickara D., Maranda E., Kaiser M., Wikramanayake T.C., Jimenez J.J. (2020). Examining the Safety and Efficacy of Low-Level Laser Therapy for Male and Female Pattern Hair Loss: A Review of the Literature. Ski. Appendage Disord..

[114] Pillai J.K., Mysore V. (2021). Role of Low-Level Light Therapy (LLLT) in Androgenetic Alopecia. J. Cutan. Aesthetic Surg..

[115] Lee G.Y., Lee S.J., Kim W.S. (2011). The effect of a 1550 nm fractional erbium-glass laser in female pattern hair loss. J. Eur. Acad. Dermatol. Venereol..

[116] Suchonwanit P., Chalermroj N., Khunkhet S. (2019). Low-level laser therapy for the treatment of androgenetic alopecia in Thai men and women: A 24-week, randomized, double-blind, sham device-controlled trial. Lasers Med. Sci..

[117] Rossi A., Campo D., Fortuna M.C., Garelli V., Pranteda G., De Vita G., Sorriso-Valvo L., Di Nunno D., Carlesimo M. (2018). A preliminary study on topical cetirizine in the therapeutic management of androgenetic alopecia. J. Dermatolog. Treat..

[118] Chen X., Xiang H., Yang M. (2022). Topical cetirizine for treating androgenetic alopecia: A systematic review. J. Cosmet Dermatol..

[119] Tsuboi R., Niiyama S., Irisawa R., Harada K., Nakazawa Y., Kishimoto J. (2020). Autologous cell-based therapy for male and female pattern hair loss using dermal sheath cup cells: A randomized placebo-controlled double-blinded dose-finding clinical study. J. Am. Acad. Dermatol..

[120] Piérard-Franchimont C., De Doncker P., Cauwenbergh G., Piérard G.E. (1998). Ketoconazole shampoo: Effect of long-term use in androgenic alopecia. Dermatology.

[121] Fischer T.W., Trüeb R.M., Hänggi G., Innocenti M., Elsner P. (2012). Topical melatonin for treatment of androgenetic alopecia. Int. J. Trichology.

[122] Nichols A.J., Hughes O.B., Canazza A., Zaiac M.N. (2017). An Open-Label Evaluator Blinded Study of the Efficacy and Safety of a New Nutritional Supplement in Androgenetic Alopecia: A Pilot Study. J. Clin. Aesthet. Dermatol..

[123] Ocampo-Garza S.S., Fabbrocini G., Ocampo-Candiani J., Cinelli E., Villani A. (2020). Micro needling: A novel therapeutic approach for androgenetic alopecia, A Review of Literature. Dermatol. Ther..

[124] McElwee K.J., Shapiro J.S. (2012). Promising therapies for treating and/or preventing androgenic alopecia. Ski. Ther. Lett..

[125] Gilhar A., Etzioni A., Paus R. (2012). Alopecia areata. N. Engl. J. Med..

[126] Rossi A., Muscianese M., Piraccini B.M., Starace M., Carlesimo M., Mandel V.D., Alessandrini A., Calvieri S., Caro G., D’Arino A. (2019). Italian Guidelines in diagnosis and treatment of alopecia areata. G. Ital. Di Dermatol. E Venereol..

[127] Mirzoyev S.A., Schrum A.G., Davis M.D.P., Torgerson R.R. (2014). Lifetime incidence risk of alopecia areata estimated at 2.1% by Rochester Epidemiology Project, 1990–2009. J. Investig. Dermatol..

[128] Safavi K.H., Muller S.A., Suman V.J., Moshell A.N., Melton L.J. (1995). Incidence of alopecia areata in Olmsted County, Minnesota, 1975 through 1989. Mayo Clin. Proc..

[129] Aranishi T., Ito T., Fukuyama M., Isaka Y., Mackie D.S., King-Concialdi K., Senglaub S.S., Jaffe D.H., Shimomura Y., Ohyama M. (2022). Prevalence of alopecia areata in Japan: Estimates from a nationally representative sample. J. Dermatol..

[130] Ito T., Ito N., Bettermann A., Tokura Y., Takigawa M., Paus R. (2004). Collapse and restoration of MHC class-I-dependent immune privilege: Exploiting the human hair follicle as a model. Am. J. Pathol..

[131] Ito T., Ito N., Saatoff M., Hashizume H., Fukamizu H., Nickoloff B.J., Takigawa M., Paus R. (2008). Maintenance of hair follicle immune privilege is linked to prevention of NK cell attack. J. Investig. Dermatol..

[132] Xing L., Dai Z., Jabbari A., Cerise J.E., Higgins C.A., Gong W., de Jong A., Harel S., DeStefano G.M., Rothman L. (2014). Alopecia areata is driven by cytotoxic T lymphocytes and is reversed by JAK inhibition. Nat. Med..

[133] King B., Ohyama M., Kwon O., Zlotogorski A., Ko J., Mesinkovska N.A., Hordinsky M., Dutronc Y., Wu W.S., McCollam J. (2022). Two Phase 3 Trials of Baricitinib for Alopecia Areata. N. Engl. J. Med..

[134] Rajabi F., Drake L.A., Senna M.M., Rezaei N. (2018). Alopecia areata: A review of disease pathogenesis. Br. J. Dermatol..

[135] Loh S.H., Moon H.N., Lew B.L., Sim W.Y. (2018). Role of T helper 17 cells and T regulatory cells in alopecia areata: Comparison of lesion and serum cytokine between controls and patients. J. Eur. Acad. Dermatol. Venereol..

[136] Fuentes-Duculan J., Gulati N., Bonifacio K.M., Kunjravia N., Zheng X., Suárez-Fariñas M., Shemer A., Guttman-Yassky E., Krueger J.G. (2016). Biomarkers of alopecia areata disease activity and response to corticosteroid treatment. Exp. Dermatol..

[137] Suárez-Fariñas M., Ungar B., Noda S., Shroff A., Mansouri Y., Fuentes-Duculan J., Czernik A., Zheng X., Estrada Y.D., Xu H. (2015). Alopecia areata profiling shows TH1, TH2, and IL-23 cytokine activation without parallel TH17/TH22 skewing. J. Allergy Clin. Immunol..

[138] Kang Y., Cai Y., Zhao Y., Yang Y. (2022). The gut microbiome and Alopecia areata: Implications for early diagnostic biomarkers and novel therapies. Front. Nutr..

[139] Ramos P.M., Anzai A., Duque-Estrada B., Melo D.F., Sternberg F., Santos L.D.N., Alves L.D., Mulinari-Brenner F. (2020). Consensus on the treatment of alopecia areata—Brazilian Society of Dermatology. An. Bras. De Dermatol..

[140] Cranwell W.C., Lai V.W., Photiou L., Meah N., Wall D., Rathnayake D., Joseph S., Chitreddy V., Gunatheesan S., Sindhu K. (2019). Treatment of alopecia areata: An Australian expert consensus statement. Australas. J. Dermatol..

[141] Messenger A.G., McKillop J., Farrant P., McDonagh A.J., Sladden M. (2012). British Association of Dermatologists’ guidelines for the management of alopecia areata 2012. Br. J. Dermatol..

[142] Meah N., Wall D., York K., Bhoyrul B., Bokhari L., Asz-Sigall D., Bergfeld W.F., Betz R.C., Blume-Peytavi U., Callender V. (2021). The Alopecia Areata Consensus of Experts (ACE) study part II: Results of an international expert opinion on diagnosis and laboratory evaluation for alopecia areata. J. Am. Acad. Dermatol..

[143] Fukuyama M., Kinoshita-Ise M., Sato Y., Ohyama M. (2020). Elucidation of demographic, clinical and trichoscopic features for early diagnosis of self-healing acute diffuse and total alopecia. J. Dermatol..

[144] Lensing M., Jabbari A. (2022). An overview of JAK/STAT pathways and JAK inhibition in alopecia areata. Front. Immunol..

[145] Kennedy Crispin M., Ko J.M., Craiglow B.G., Li S., Shankar G., Urban J.R., Chen J.C., Cerise J.E., Jabbari A., Winge M.C. (2016). Safety and efficacy of the JAK inhibitor tofacitinib citrate in patients with alopecia areata. JCI Insight.

[146] Mackay-Wiggan J., Jabbari A., Nguyen N., Cerise J.E., Clark C., Ulerio G., Furniss M., Vaughan R., Christiano A.M., Clynes R. (2016). Oral ruxolitinib induces hair regrowth in patients with moderate-to-severe alopecia areata. JCI Insight.

[147] King B., Guttman-Yassky E., Peeva E., Banerjee A., Sinclair R., Pavel A.B., Zhu L., Cox L.A., Craiglow B., Chen L. (2021). A phase 2a randomized, placebo-controlled study to evaluate the efficacy and safety of the oral Janus kinase inhibitors ritlecitinib and brepocitinib in alopecia areata: 24-week results. J. Am. Acad. Dermatol..

[148] Jabbari A., Sansaricq F., Cerise J., Chen J.C., Bitterman A., Ulerio G., Borbon J., Clynes R., Christiano A.M., Mackay-Wiggan J. (2018). An Open-Label Pilot Study to Evaluate the Efficacy of Tofacitinib in Moderate to Severe Patch-Type Alopecia Areata, Totalis, and Universalis. J. Investig. Dermatol..

[149] Mikhaylov D., Glickman J.W., Del Duca E., Nia J., Hashim P., Singer G.K., Posligua A.L., Florek A.G., Ibler E., Hagstrom E.L. (2023). A phase 2a randomized vehicle-controlled multi-center study of the safety and efficacy of delgocitinib in subjects with moderate-to-severe alopecia areata. Arch. Dermatol. Res..

[150] King B., Mesinkovska N., Mirmirani P., Bruce S., Kempers S., Guttman-Yassky E., Roberts J.L., McMichael A., Colavincenzo M., Hamilton C. (2022). Phase 2 randomized, dose-ranging trial of CTP-543, a selective Janus Kinase inhibitor, in moderate-to-severe alopecia areata. J. Am. Acad. Dermatol..

[151] Guo L., Feng S., Sun B., Jiang X., Liu Y. (2020). Benefit and risk profile of tofacitinib for the treatment of alopecia areata: A systemic review and meta-analysis. J. Eur. Acad. Dermatol. Venereol..

[152] McKenzie P.L., Castelo-Soccio L. (2021). Alopecia areata flare patterns in children and young adults while on systemic tofacitinib. J. Am. Acad. Dermatol..

[153] Harada K., Irisawa R., Ito T., Uchiyama M., Tsuboi R. (2020). The effectiveness of dupilumab in patients with alopecia areata who have atopic dermatitis: A case series of seven patients. Br. J. Dermatol..

[154] Uchida H., Kamata M., Watanabe A., Agematsu A., Nagata M., Fukaya S., Hayashi K., Fukuyasu A., Tanaka T., Ishikawa T. (2019). Dupilumab Improved Alopecia Areata in a Patient with Atopic Dermatitis: A Case Report. Acta Derm. Venereol..

[155] Ushida M., Ohshita A., Arakawa Y., Kanehisa F., Katoh N., Asai J. (2020). Dupilumab therapy rapidly improved alopecia areata associated with trichotillomania in an atopic dermatitis patient. Allergol. Int..

[156] Guttman-Yassky E., Renert-Yuval Y., Bares J., Chima M., Hawkes J.E., Gilleaudeau P., Sullivan-Whalen M., Singer G.K., Garcet S., Pavel A.B. (2022). Phase 2a randomized clinical trial of dupilumab (anti-IL-4Rα) for alopecia areata patients. Allergy.

[157] Yoshimasu T., Uede M., Kanazawa N., Mikita N., Yamamoto Y., Ito T., Furukawa F. (2014). Involvement of FcɛR1α immunopositive cells in alopecia areata with atopic dermatitis and a high titer of serum immunoglobulin E. Eur. J. Dermatol..

[158] Renert-Yuval Y., Pavel A.B., Del Duca E., Facheris P., Pagan A.D., Bose S., Gómez-Arias P.J., Angelov M., Bares J., Chima M. (2022). Scalp Biomarkers During Dupilumab Treatment Support Th2 Pathway Pathogenicity in Alopecia Areata. Allergy.

[159] Chung J., Slaught C.L., Simpson E.L. (2019). Alopecia areata in 2 patients treated with dupilumab: New onset and worsening. JAAD Case Rep..

[160] Kageyama R., Ito T., Hanai S., Morishita N., Nakazawa S., Fujiyama T., Honda T., Tokura Y. (2021). Immunological Properties of Atopic Dermatitis-Associated Alopecia Areata. Int. J. Mol. Sci..

[161] Almohanna H.M., Ahmed A.A., Griggs J.W., Tosti A. (2020). Platelet-Rich Plasma in the Treatment of Alopecia Areata: A Review. J. Investig. Dermatol. Symp. Proc..

[162] Meznerics F.A., Illés K., Dembrovszky F., Fehérvári P., Kemény L.V., Kovács K.D., Wikonkál N.M., Csupor D., Hegyi P., Bánvölgyi A. (2022). Platelet-Rich Plasma in Alopecia Areata-A Steroid-Free Treatment Modality: A Systematic Review and Meta-Analysis of Randomized Clinical Trials. Biomedicines.

[163] Balakrishnan A., Joy B., Thyvalappil A., Mathew P., Sreenivasan A., Sridharan R. (2020). A Comparative Study of Therapeutic Response to Intralesional Injections of Platelet-Rich Plasma Versus Triamcinolone Acetonide in Alopecia Areata. Indian Derm. Online J..

[164] Hegde P., Relhan V., Sahoo B., Garg V.K. (2020). A randomized, placebo and active controlled, split scalp study to evaluate the efficacy of platelet-rich plasma in patchy alopecia areata of the scalp. Dermatol. Ther..

[165] Kapoor P., Kumar S., Brar B.K., Kukar N., Arora H., Brar S.K. (2020). Comparative Evaluation of Therapeutic Efficacy of Intralesional Injection of Triamcinolone Acetonide versus Intralesional Autologous Platelet-rich Plasma Injection in Alopecia Areata. J. Cutan. Aesthetic Surg..

[166] Cervantes J., Jimenez J.J., DelCanto G.M., Tosti A. (2018). Treatment of Alopecia Areata with Simvastatin/Ezetimibe. J. Investig. Dermatol. Symp. Proc..

[167] Shin J.M., Jung K.E., Yim S.H., Rao B., Hong D., Seo Y.J., Kim C.D., Lee Y. (2021). Putative therapeutic mechanisms of simvastatin in the treatment of alopecia areata. J. Am. Acad. Dermatol..

[168] Gherardini J., Rivas K.E., Chéret J., Strbo N., Paus R. (2022). Downregulation of pathogenic MICA-NKG2D interactions as a novel strategy in alopecia areata management: A new rationale for adjunct statin therapy?. J. Eur. Acad. Dermatol. Venereol..

[169] Lattouf C., Jimenez J.J., Tosti A., Miteva M., Wikramanayake T.C., Kittles C., Herskovitz I., Handler M.Z., Fabbrocini G., Schachner L.A. (2015). Treatment of alopecia areata with simvastatin/ezetimibe. J. Am. Acad. Dermatol..

[170] Gorcey L., Gordon Spratt E.A., Leger M.C. (2014). Alopecia universalis successfully treated with adalimumab. JAMA Dermatol..

[171] Skurkovich S., Korotky N.G., Sharova N.M., Skurkovich B. (2005). Treatment of alopecia areata with anti-interferon-gamma antibodies. J. Investig. Dermatol. Symp. Proc..

[172] Price V.H., Hordinsky M.K., Olsen E.A., Roberts J.L., Siegfried E.C., Rafal E.S., Korman N.J., Altrabulsi B., Leung H.M., Garovoy M.R. (2008). Subcutaneous efalizumab is not effective in the treatment of alopecia areata. J. Am. Acad. Dermatol..

[173] Strober B.E., Siu K., Alexis A.F., Kim G., Washenik K., Sinha A., Shupack J.L. (2005). Etanercept does not effectively treat moderate to severe alopecia areata: An open-label study. J. Am. Acad. Dermatol..

[174] Mikhaylov D., Pavel A., Yao C., Kimmel G., Nia J., Hashim P., Vekaria A.S., Taliercio M., Singer G., Karalekas R. (2019). A randomized placebo-controlled single-center pilot study of the safety and efficacy of apremilast in subjects with moderate-to-severe alopecia areata. Arch. Dermatol. Res..

[175] Trüeb R.M. (2016). Telogen Effluvium: Is There a Need for a New Classification?. Ski. Appendage Disord..

[176] Kligman A.M. (1961). Pathologic dynamics of human hair loss. I. Telogen effuvium. Arch. Dermatol..

[177] Grover C., Khurana A. (2013). Telogen effluvium. Indian J. Dermatol. Venereol. Leprol..

[178] Malkud S. (2015). Telogen Effluvium: A Review. J. Clin. Diagn Res..

[179] Headington J.T. (1993). Telogen effluvium. New concepts and review. Arch. Dermatol..

[180] Rebora A. (2016). Proposing a Simpler Classification of Telogen Effluvium. Ski. Appendage Disord..

[181] Whiting D.A. (1996). Chronic telogen effluvium. Dermatol. Clin..

[182] Seleit I., Bakry O.A., Badr E., Hassan E.H. (2019). Vitamin D Receptor Gene Polymorphism In Chronic Telogen Effluvium; A Case-Control Study. Clin. Cosmet. Investig. Dermatol..

[183] Bernstein G.M., Crollick J.S., Hassett J.M. (1988). Postfebrile telogen effluvium in critically ill patients. Crit. Care Med..

[184] Ohyama M., Matsudo K., Fujita T. (2022). Management of hair loss after severe acute respiratory syndrome coronavirus 2 infection: Insight into the pathophysiology with implication for better management. J. Dermatol..

[185] Starace M., Piraccini B.M., Evangelista V., Bruni F., Alessandrini A. (2023). Acute telogen effluvium due to Dengue fever mimicking androgenetic alopecia. Ital. J. Dermatol. Venerol..

[186] Trüeb R.M. (2010). Systematic approach to hair loss in women. J. Der Dtsch. Dermatol. Ges..

[187] Antonelli M., Pujol J.C., Spector T.D., Ourselin S., Steves C.J. (2022). Risk of long COVID associated with delta versus omicron variants of SARS-CoV-2. Lancet.

[188] Hoffmann M., Kleine-Weber H., Schroeder S., Kruger N., Herrler T., Erichsen S., Schiergens T.S., Herrler G., Wu N.H., Nitsche A. (2020). SARS-CoV-2 Cell Entry Depends on ACE2 and TMPRSS2 and Is Blocked by a Clinically Proven Protease Inhibitor. Cell.

[189] Goren A., Wambier C.G., Herrera S., McCoy J., Vaño-Galván S., Gioia F., Comeche B., Ron R., Serrano-Villar S., Ramos P.M. (2021). Anti-androgens may protect against severe COVID-19 outcomes: Results from a prospective cohort study of 77 hospitalized men. J. Eur. Acad. Dermatol. Venereol..

[190] Montopoli M., Zumerle S., Vettor R., Rugge M., Zorzi M., Catapano C.V., Carbone G.M., Cavalli A., Pagano F., Ragazzi E. (2020). Androgen-deprivation therapies for prostate cancer and risk of infection by SARS-CoV-2: A population-based study (N = 4532). Ann. Oncol..

[191] Kim J., Hong K., Gómez Gómez R.E., Kim S., Chun B.C. (2021). Lack of Evidence of COVID-19 Being a Risk Factor of Alopecia Areata: Results of a National Cohort Study in South Korea. Front. Med..

[192] Ganjei Z., Yazdan Panah M., Rahmati R., Zari Meidani F., Mosavi A. (2022). COVID-19 vaccination and alopecia areata: A case report and literature review. Clin. Case Rep..

[193] Michelini S., Caro G., Di Fraia M., Fortuna M., Magri F., Gomes V.V., Grieco T., Carlesimo M., Rossi A., Pellacani G. (2023). Telogen effluvium in SARS-CoV-2 infection: Histological aspects. J. Eur. Acad. Dermatol. Venereol..

[194] Senna M.M., Peterson E., Jozic I., Chéret J., Paus R. (2022). Frontiers in Lichen Planopilaris and Frontal Fibrosing Alopecia Research: Pathobiology Progress and Translational Horizons. JID Innov..

[195] Harries M., Hardman J., Chaudhry I., Poblet E., Paus R. (2020). Profiling the human hair follicle immune system in lichen planopilaris and frontal fibrosing alopecia: Can macrophage polarization differentiate these two conditions microscopically?. Br. J. Dermatol..

[196] Wang E.H.C., Monga I., Sallee B.N., Chen J.C., Abdelaziz A.R., Perez-Lorenzo R., Bordone L.A., Christiano A.M. (2022). Primary cicatricial alopecias are characterized by dysregulation of shared gene expression pathways. PNAS Nexus.

[197] Pavlovsky L., Israeli M., Sagy E., Berg A.L., David M., Shemer A., Klein T., Hodak E. (2015). Lichen planopilaris is associated with HLA DRB1*11 and DQB1*03 alleles. Acta Derm. Venereol..

[198] Tziotzios C., Petridis C., Dand N., Ainali C., Saklatvala J.R., Pullabhatla V., Onoufriadis A., Pramanik R., Baudry D., Lee S.H. (2019). Genome-wide association study in frontal fibrosing alopecia identifies four susceptibility loci including HLA-B*07:02. Nat Commun.

[199] Cuenca-Barrales C., Ruiz-Villaverde R., Molina-Leyva A. (2021). Familial Frontal Fibrosing Alopecia: Report of a case and systematic review of the literature. Sultan Qaboos Univ. Med. J..

[200] Dlova N., Goh C.L., Tosti A. (2013). Familial frontal fibrosing alopecia. Br. J. Dermatol..

[201] Ranasinghe G.C., Piliang M.P., Bergfeld W.F. (2017). Prevalence of hormonal and endocrine dysfunction in patients with lichen planopilaris (LPP): A retrospective data analysis of 168 patients. J. Am. Acad. Dermatol..

[202] Kinoshita-Ise M., Fukuyama M., Ohyama M. (2022). Distinctive age distribution and hair loss pattern putatively highlighting uniqueness of Japanese cases of fibrosing alopecia in a pattern distribution. J. Dermatol..

[203] Malki L., Sarig O., Romano M.T., Méchin M.C., Peled A., Pavlovsky M., Warshauer E., Samuelov L., Uwakwe L., Briskin V. (2019). Variant PADI3 in Central Centrifugal Cicatricial Alopecia. N. Engl. J. Med..

[204] Strowd L.C., Subash J., McGregor S., McMichael A. (2018). Cicatricial Alopecia in Identical Twin Lumbee Native American Women. Ski. Appendage Disord..

[205] Kyei A., Bergfeld W.F., Piliang M., Summers P. (2011). Medical and environmental risk factors for the development of central centrifugal cicatricial alopecia: A population study. Arch. Dermatol..

[206] Dina Y., Okoye G.A., Aguh C. (2018). Association of Uterine Leiomyomas With Central Centrifugal Cicatricial Alopecia. JAMA Dermatol..

[207] Jamerson T.A., Talbot C.C., Dina Y., Aguh C. (2022). Presence of Uterine Leiomyomas Has No Significant Impact on Gene Expression Profile in the Scalp of Patients with Central Centrifugal Cicatricial Alopecia. JID Innov..

[208] Olsen E.A., Bergfeld W.F., Cotsarelis G., Price V.H., Shapiro J., Sinclair R., Solomon A., Sperling L., Stenn K., Whiting D.A. (2003). Summary of North American Hair Research Society (NAHRS)-sponsored Workshop on Cicatricial Alopecia, Duke University Medical Center, February 10 and 11, 2001. J. Am. Acad. Dermatol..

[209] Yip L., Barrett T.H., Harries M.J. (2020). Folliculitis decalvans and lichen planopilaris phenotypic spectrum: A case series of biphasic clinical presentation and theories on pathogenesis. Clin. Exp. Dermatol..

[210] Egger A., Stojadinovic O., Miteva M. (2020). Folliculitis Decalvans and Lichen Planopilaris Phenotypic Spectrum-A Series of 7 New Cases With Focus on Histopathology. Am. J. Dermatopathol..

[211] Matard B., Cavelier Balloy B., Assouly P., Reygagne P. (2021). It has the Erythema of a Lichen Planopilaris, it has the Hyperkeratosis of a Lichen Planopilaris, but it is Not a Lichen Planopilaris: About the "Lichen Planopilaris-Like" Form of Folliculitis Decalvans. Am. J. Dermatopathol..

[212] Shavit E., Cohen A., Zoller L., Onn E., Kridin K. (2023). The burden of gout in acne keloidalis nuchae-Insights from a population-based study. J. Cosmet. Dermatol..

[213] Umar S., Lullo J.J., Carter M.J., Shitabata P.K., Lee D.J. (2022). Acne Keloidalis Nuchae is Associated with Cutis Verticis Gyrata. Clin. Cosmet. Investig. Dermatol..

[214] Zagelbaum Ward N.K., Jun J.A., Vecerek N., Donaldson M., Quismorio F.P. (2022). Dissecting cellulitis of the scalp associated with peripheral and axial spondyloarthritis: Report of a case and review of the literature. Clin. Rheumatol..

[215] Thomas J., Aguh C. (2021). Approach to treatment of refractory dissecting cellulitis of the scalp: A systematic review. J. Dermatolog. Treat..

[216] Iorizzo M., Starace M., Vano-Galvan S., Piraccini B.M., Reygagne P., Rudnicka L., Silyuk T., Sinclair R., Tosti A. (2022). Refractory folliculitis decalvans treated with adalimumab: A case series of 23 patients. J. Am. Acad. Dermatol..

[217] Rambhia P.H., Conic R.R.Z., Murad A., Atanaskova-Mesinkovska N., Piliang M., Bergfeld W. (2019). Updates in therapeutics for folliculitis decalvans: A systematic review with evidence-based analysis. J. Am. Acad. Dermatol..

[218] Mesinkovska N.A., Tellez A., Dawes D., Piliang M., Bergfeld W. (2015). The use of oral pioglitazone in the treatment of lichen planopilaris. J. Am. Acad. Dermatol..

[219] Mirmirani P., Karnik P. (2009). Lichen planopilaris treated with a peroxisome proliferator-activated receptor gamma agonist. Arch. Dermatol..

[220] Peterson E.L., Gutierrez D., Brinster N.K., Lo Sicco K.I., Shapiro J. (2019). Response of Lichen Planopilaris to Pioglitazone Hydrochloride. J. Drugs Dermatol..

[221] Spring P., Spanou Z., de Viragh P.A. (2013). Lichen planopilaris treated by the peroxisome proliferator activated receptor-γ agonist pioglitazone: Lack of lasting improvement or cure in the majority of patients. J. Am. Acad. Dermatol..

[222] Lajevardi V., Ghiasi M., Balighi K., Daneshpazhooh M., Azar P.M., Kianfar N., Dasdar S., Peymanfar A.A. (2022). Efficacy and safety of oral pioglitazone in the management of lichen planopilaris in comparison with clobetasol: A randomized clinical trial. Dermatol. Ther..

[223] Yang C.C., Khanna T., Sallee B., Christiano A.M., Bordone L.A. (2018). Tofacitinib for the treatment of lichen planopilaris: A case series. Dermatol. Ther..

[224] Plante J., Eason C., Snyder A., Elston D. (2020). Tofacitinib in the treatment of lichen planopilaris: A retrospective review. J. Am. Acad. Dermatol..

[225] Moussa A., Bhoyrul B., Asfour L., Kazmi A., Eisman S., Sinclair R.D. (2022). Treatment of lichen planopilaris with baricitinib: A retrospective study. J. Am. Acad. Dermatol..

[226] Shao S., Tsoi L.C., Sarkar M.K., Xing X., Xue K., Uppala R., Berthier C.C., Zeng C., Patrick M., Billi A.C. (2019). IFN-γ enhances cell-mediated cytotoxicity against keratinocytes via JAK2/STAT1 in lichen planus. Sci. Transl. Med..

[227] Alzahrani M., Coste V., Konstantinou M.P., Reguiai Z., Villani A., Hotz C., Viguier M., Pruvost-Balland C., Dupuy A., Wolkenstein P. (2023). Treatment of dissecting cellulitis of the scalp with tumor necrosis factor inhibitors: A retrospective multicenter study. Clin. Exp. Dermatol..

[228] Sobell J.M. (2016). Update on TNF Inhibitors in Dermatology. Semin. Cutan. Med. Surg..

